# Epigenetic drugs in somatostatin type 2 receptor radionuclide theranostics and radiation transcriptomics in mouse pheochromocytoma models

**DOI:** 10.7150/thno.77918

**Published:** 2023-01-01

**Authors:** Martin Ullrich, Susan Richter, Josephine Liers, Stephan Drukewitz, Markus Friedemann, Jörg Kotzerke, Christian G. Ziegler, Svenja Nölting, Klaus Kopka, Jens Pietzsch

**Affiliations:** 1 Helmholtz-Zentrum Dresden-Rossendorf, Institute of Radiopharmaceutical Cancer Research, Department of Radiopharmaceutical and Chemical Biology, Dresden, Germany.; 2 University Hospital Carl Gustav Carus at the Technische Universität Dresden, Institute of Clinical Chemistry and Laboratory Medicine, Dresden, Germany.; 3 National Center for Tumor Diseases/University Cancer Center Dresden, Core Unit for Molecular Tumor Diagnostics, Dresden, Germany.; 4 University of Leipzig Medical Center, Institute of Human Genetics, Leipzig, Germany.; 5 University Hospital Carl Gustav Carus at the Technische Universität Dresden, Klinik und Poliklinik für Nuklearmedizin, Dresden, Germany.; 6 University Hospital Carl Gustav Carus at the Technische Universität Dresden, Department of Medicine III, Dresden, Germany.; 7 University Hospital Zurich (USZ) and University of Zurich (UZH), Department of Endocrinology, Diabetology and Clinical Nutrition, Zurich, Switzerland.; 8 University Hospital, LMU Munich, Department of Medicine IV, Munich, Germany.; 9 Technische Universität Dresden, School of Science, Faculty of Chemistry and Food Chemistry, Dresden, Germany.; 10 German Cancer Consortium (DKTK), Partner Site Dresden, Dresden, Germany.; 11 National Center for Tumor Diseases (NCT), Partner Site Dresden, University Cancer Center (UCC), Dresden, Germany.

**Keywords:** decitabine, neuroendocrine tumors, radiation biology, small animal multimodal imaging, valproic acid

## Abstract

Pheochromocytomas and paragangliomas (PCCs/PGLs) are catecholamine-producing tumors. In inoperable and metastatic cases, somatostatin type 2 receptor (SSTR2) expression allows for peptide receptor radionuclide therapy with [^177^Lu]Lu-DOTA-TATE. Insufficient receptor levels, however, limit treatment efficacy. This study evaluates whether the epigenetic drugs valproic acid (VPA) and *5*-Aza-*2'*-deoxycytidine (DAC) modulate SSTR2 levels and sensitivity to [^177^Lu]Lu-DOTA-TATE in two mouse PCC models (MPC and MTT).

**Methods:** Drug-effects on *Sstr2*/SSTR2 were investigated in terms of promoter methylation, mRNA and protein levels, and radiotracer binding. Radiotracer uptake was measured in subcutaneous allografts in mice using PET and SPECT imaging. Tumor growth and gene expression (RNAseq) were characterized after drug treatments.

**Results:** DAC alone and in combination with VPA increased SSTR2 levels along with radiotracer uptake *in vitro* in MPC (*high-*SSTR2) and MTT cells (*low-*SSTR2). MTT but not MPC allografts responded to DAC and VPA combination with significantly elevated radiotracer uptake, although activity concentrations remained far below those in MPC tumors. In both models, combination of DAC, VPA and [^177^Lu]Lu-DOTA-TATE was associated with additive effects on tumor growth delay and specific transcriptional responses in gene sets involved in cancer and treatment resistance. Effects of epigenetic drugs were unrelated to CpG island methylation of the *Sstr2* promoter.

**Conclusion:** This study demonstrates that SSTR2 induction in mouse pheochromocytoma models has some therapeutic benefit that occurs via yet unknown mechanisms. Transcriptional changes in tumor allografts associated with epigenetic treatment and [^177^Lu]Lu-DOTA-TATE provide first insights into genetic responses of PCCs/PGLs, potentially useful for developing additional strategies to prevent tumor recurrence.

## Introduction

Adrenal pheochromocytomas and extra-adrenal paragangliomas (PCCs/PGLs) are rare catecholamine-producing neuroendocrine tumors arising from neural crest-derived chromaffin tissue 1. About 70‒80% of these tumors are associated with inherited or somatic mutations in one of over 20 currently identified PCC/PGL susceptibility genes 2, 3. Most PCCs/PGLs are benign, however, metastatic disease occurs in about 10% of PCCs and in 35‒40% of PGLs, in particular associated with mutations in succinate dehydrogenase A and B genes 4-7.

Endoradiotherapy targeting norepinephrine transporters with high-molar activity *meta-*[^131^I]iodobenzylguanidine (Ultratrace®) is currently the only officially (FDA) approved treatment option for metastatic PCCs/PGLs in the US 8. Since high somatostatin type 2 receptor (SSTR2) levels can be found in roughly half of all PCC/PGLs 9, peptide receptor radionuclide therapy (PRRT) using ^177^Lu- and ^90^Y-labeled somatostatin analogs has also been increasingly taken into account as an effective treatment for metastatic PCCs/PGLs 4, 10. In particular PRRT using [^177^Lu]Lu-DOTA-TATE (Lutathera®) is one of the most promising treatment options and has been approved for gastro-enteropancreatic and pulmonary neuroendocrine tumors in many countries 11.

Two small studies applying PRRT to patients suffering from PCCs/PGLs showed partial responses in 30-60% and disease stabilization in 71-100% of cases 10, 12. Average progression-free survival of 38 months and overall survival of 60 months have been reported with [^177^Lu]Lu-DOTA-TATE 13.

All radionuclide therapies rely on sufficient uptake of the radiopharmaceutical by tumor cells, with higher uptake correlating to increased treatment response 14. Hence, high SSTR2 levels are important for PRRT in neuroendocrine tumors to ensure efficient delivery of radiation. SSTR2 levels differ between patients with neuroendocrine tumors, including PCCs/PGLs, and are a limiting factor for treatment efficacy 12, 15, 16.

Both DNA methylation and histone acetylation might be involved in regulating *SSTR2* gene expression, i.e. modulating heterochromatin and euchromatin accessibility, respectively. Successful up-regulation of SSTR2 was demonstrated in various neuroendocrine tumor cell lines and tumor models through attenuation of DNA methylation by treatment with DNA-*N*-methyltransferase (DNMT) inhibitors and via increase in histone acetylation levels by treatment with histone deacetylase (HDAC) inhibitors 16-27. It was proposed that *SSTR2* expression is regulated by DNA methylation of a CpG island within the *SSTR2* promoter 20. Moreover, it was suggested that epigenetic drugs might sensitize tumor cells to other treatments 28 and allow use of reduced, less toxic, doses for these other agents.

Mouse PCC cell lines are frequently used as PCC/PGL models for preclinical research 29, 30. Mouse pheochromocytoma (MPC) cells originate from an adrenal tumor of a neurofibromin 1-knockout mouse and mirror human disease in terms of catecholamine production and high SSTR2 levels 9, 31, 32. Subcutaneous and metastasized MPC allograft models were developed in various mouse strains 30, 33-37. Building upon this, the mouse tumor tissue-derived (MTT) cell line was generated from an MPC liver metastasis resulting in a more aggressive phenotype in terms of tumor progression and metastatic spread 38. Both models are suitable for preclinical testing of new PCC/PGL therapies 30, and similar to human *SSTR2* the mouse *Sstr2* gene has a CpG island located upstream of the promoter.

The present study addresses the hypothesis that epigenetic treatment of PCC/PGL cell and tumor models with the HDAC inhibitor valproic acid (VPA) and the DNMT inhibitor *5*-Aza-*2'*-deoxycytidine (DAC) is associated with (1) up-regulation of SSTR2, (2) increased uptake of radiolabeled somatostatin analogs, and (3) neo-adjuvant effects in combination with PRRT. The objectives of this study are (i) to establish an appropriate dose regimen for epigenetic treatment with VPA and DAC allowing for inducing both SSTR2 levels and uptake of SSTR2 radiotracers, such as [^68^Ga]Ga- and [^64^Cu]-Cu-DOTA-TATE, in MPC and MTT cells, (ii) to evaluate the effects of epigenetic treatment on tumor uptake of SSTR2 radiotracers in MPC and MTT allograft mice, and (iii) to characterize combination effects of epigenetic drugs and [^177^Lu]Lu-DOTA-TATE on growth response and gene expression of MPC and MTT tumors.

## Materials and methods

### Cells and substances

MPC (passages 35-40) and MTT cells (passages 22-28) were routinely cultured as described elsewhere 33. Epigenetic drugs VPA and DAC were purchased from Sigma-Aldrich (St. Louis, MO, USA). Viability assay reagent *3*-(*4,5*-dimethylthiazol-*2*-yl)-*2,5*-diphenyltetrazolium bromide was purchased from Thermo Fischer Scientific (Waltham, MA, USA).

### Epigenetic treatment *in vitro*

Cells were seeded into collagen-coated microplates or collagen-coated flasks 24 h before epigenetic treatment (ET) start and treated twice (24 h interval) with VPA and DAC, as single and combination doses, respectively. Treatment effects were investigated two to six days (d) after final ET. Preparation of epigenetic drugs is reported in *Supplemental Information 1.1*.

### Viability assay

Viability of cells seeded into collagen-coated 96-well microplates (6×10^4^/cm^2^) was measured three days after treatment start using a colorimetric tetrazolium dye assay 39. Doxorubicin served as positive control.

### Immunoblotting

Cells seeded into collagen-coated flasks (2.5×10^4^/cm^2^) were harvested three days after treatment start by incubation with Dulbecco's phosphate-buffered saline containing 2 mmol/L EDTA at 4 °C. Cell pellets were frozen and stored at -70°C. Frozen tumor tissue obtained from allograft mice was cut into 4 × 4 mm pieces.

Protein extraction from cells and tissues, gel electrophoresis, and immunoblotting was performed as described previously 34 with some modifications. In brief, tissue homogenization was performed using the gentleMACS™ dissociator (Miltenyi Biotech, Bergisch Gladbach, Germany). Immunodetection of proteins was performed using the recombinant primary antibodies anti-SSTR2 [UMB1] (Abcam, Cambridge, UK), anti-CHGA [C-20] (Santa Cruz Biotechnology, Heidelberg, Germany), and anti-ACTB [A5316] (Sigma-Aldrich). Densitometric analysis of protein bands was performed using ImageQuant TL (GE Healthcare, Chicago, IL, USA).

### Radiolabeling of DOTA-TATE

Radionuclide production and supply are described in *Supplemental Information 1.2*. Radiolabeling of DOTA-TATE (Bachem, Bubendorf, Switzerland) was performed as described previously 34, resulting in radiochemical yields of 95-98% for [^68^Ga]Ga-DOTA-TATE, 97-99% for [^64^Cu]Cu-DOTA-TATE, and 95-99% for [^177^Lu]Lu-DOTA-TATE as determined using analytical radio-high performance liquid chromatography (radio-HPLC).

### Radioligand uptake in cells

Cells were seeded into collagen-coated 24-well microplates (2.5×10^4^/cm^2^) and uptake of [^68^Ga]Ga-DOTA-TATE was measured three and seven days after ET start. Cells were incubated with fresh cell culture medium containing 0.8 MBq/mL [^68^Ga]Ga-DOTA-TATE (A_m_ = 10 MBq/nmol), equivalent to 80 nmol/L, for 60 min at 4 °C or 37 °C. Cells were washed and lysed, and activity of cell lysates was measured as described previously 34. Total protein in cell lysates was measured using the Qubit protein assay (Thermo Fisher Scientific). Uptake of [^68^Ga]Ga-DOTA-TATE was reported as % initial dose per mg of protein.

### SSTR2 saturation binding assay

SSTR2 saturation binding was measured as previously described 40, with some modifications. In brief, cells were seeded into collagen-coated flasks (5×10^4^/cm^2^) and harvested three days after ET start by incubation with Dulbecco's phosphate-buffered saline containing 2 mmol/L EDTA at 4 °C. Cells were resuspended and frozen in fetal bovine serum containing 10% (v/v) of DMSO and stored at -70 °C. Cell homogenates were incubated with [^64^Cu]Cu-DOTA-TATE (A_m_ = 70 MBq/nmol) at final concentrations between 0.625 and 10 nmol/L. Dissociation constants (*K*_d_) and maximum binding capacities (*B*_max_) were calculated by fitting the one-site specific binding equation using Prism 8.0 (GraphPad Software, San Diego, CA, USA). Cell numbers in final samples were re-calculated from protein concentrations (conversion through linear regression between cell number and protein concentration).

### Animal experiment

All animal experiments were carried out according to the guidelines of the German Regulations for Animal Welfare and have been approved by the local Ethical Committee for Animal Experiments. A number of 4×10^6^ MPC or MTT cells were re-suspended in 40 µL of Dulbecco's phosphate-buffered saline and injected subcutaneously into the shoulder of 10-15 week-old female nude mice (Rj:NMRI-Foxn1^nu/nu^, Janvier Labs, Le Genest-Saint-Isle, France). Prior to imaging procedures, general anesthesia was induced and maintained with inhalation of 10% (v/v) desflurane in 30/10% (v/v) oxygen/air. During anesthesia, animals were continuously warmed at 37°C. Tumor growth was monitored three times per week using caliper measurements. Tumor volume was calculated assuming a tri-axial ellipsoid with the axes *a*, *b*, and *c* using the formula V = *π*/6×*abc*. Animals were sacrificed using CO_2_ inhalation and cervical dislocation.

### Epigenetic treatment of mice

Preparation of epigenetic drugs is described in *Supplemental Information 1.1*. Animals received VPA (1.7×10^‒3^ mol/kg ≈ 250 mg/kg), DAC (4.4×10^‒9^ mol/kg ≈ 1 mg/kg), or a combination of both, in Dulbecco's phosphate buffered saline (200 µL/30 g body weight), delivered as two consecutive intraperitoneal injections in a 72 h-interval. Applied doses were within the epigenetically effective dose range in mice, as reported elsewhere 17, 41-43. The estimated human equivalent doses corresponded to 20‒30% of the clinically applied tumor-suppressive doses in patients 44, 45. Animals in control groups received Dulbecco's phosphate buffered saline only. Multiple independent experiments were performed, always including animals of all treatment groups (Table 1). Tumor volumes in treatment groups at the beginning of ET and PRRT are summarized in *Supplemental Information 1.3.*

### Small animal PET/CT imaging

Small animal positron emission tomography (PET) was performed using the nanoPET/CT scanner (Mediso Medical Imaging Systems, Budapest, Hungary). Each animal received an intravenous injection of 10 MBq [^64^Cu]Cu-DOTA-TATE (pharmaceutically equivalent to 0.25 nmol) delivered in 0.2 mL of 0.154 mol/L NaCl(aq) through a tail vein catheter. Recording, binning, framing, and image reconstruction were performed as reported previously 34. Dynamic imaging was performed for 60 min, static imaging between 40‒60 min after radiotracer injection. With each PET scan, a corresponding CT image was recorded and used for anatomical referencing and attenuation correction. Images were post-processed and analyzed using ROVER (ABX, Radeberg, Germany) and displayed as maximum intensity projections (MIPs) at indicated time points and scaling. Standardized uptake values (SUV) were determined and reported as SUVmean (VOI-averaged) and SUVmax (VOI-maximum). Details on quantitative PET image analysis are provided in *Supplemental Information 1.4*.

### Biodistribution

Radiotracer was injected as described for PET imaging. Mice were sacrificed 60 min after radiotracer injection, organs were excised and weighted, and activity in tissue samples was measured using the gamma counter Wizard (PerkinElmer, Waltham, MA, USA). Standardized uptake values were determined.

### PRRT and small-animal SPECT/CT imaging

For initiation of peptide receptor radionuclide therapy (PRRT), each animal received a single intravenous injection of 70 MBq [^177^Lu]Lu-DOTA-TATE (pharmaceutically equivalent to 1.2 nmol) delivered in 0.2 mL of 0.154 mol/L NaCl(aq) through a tail vein catheter. Small animal single-photon emission computed tomography (SPECT) was initiated 22‒26 hours after radiotracer injection using the nanoSPECT/CT scanner (Mediso Medical Imaging Systems). With each SPECT scan, a corresponding CT image was recorded and used for anatomical referencing and attenuation correction. Images were post-processed and analyzed using ROVER (ABX) and displayed as maximum intensity projections at indicated scaling. Activity concentration in tissues was determined and reported as A*_V_* mean (VOI-averaged volume activity A*_V_*_ tissue 24h_, MBq/mL). Details on quantitative SPECT image analysis are provided in *Supplemental Information 1.5*.

### Sstr2 promoter methylation analysis

Methylation analysis was performed by bisulfite-converted DNA amplification as described in *Supplemental Information 1.6*.

### RNA sequencing

mRNA was extracted using the miRNeasy Mini Kit (Qiagen) and quality was assessed using the 5200 Fragment Analyzer System (Agilent, Santa Clara, CA, USA). For library preparation, TruSeq Stranded mRNA Library Prep Kit (Illumina Inc, San Diego, CA, USA) according to the manufacturer's protocol was used, starting with 1 µg total RNA. All barcoded libraries were pooled and sequenced 2 × 75 bp paired-end on an Illumina NextSeq 550 platform to obtain a minimum of 10 × 10^6^ reads per sample. The data obtained were made publicly available (BioProject: PRJNA866298; 'unbh' corresponds to [Control]; 'epionly' corresponds to [ET]; 'ctrl' corresponds to [PRRT]; 'kombi' corresponds to [ET + PRRT]).

### Bioinformatics analysis

Within the framework of the bioinformatic workflow, raw reads were trimmed using trimmomatic 46 and aligned using STAR 47, GRCm38 was used as reference genome. Read counts were extracted from the alignments using the featureCounts method of the subread package 48; afterwards, DESeq2 was applied to identify differentially expressed genes 49. Differences in gene expression with multiple testing adjusted *P*-values (padj from DESeq2) < 0.05 were considered significant. Differentially expressed genes showing a *log_2_* fold change (l2fc) > |1| and a standard error l2fc < 0.5 were reported. Heatmaps of fragments per kilobase million (fpkm) z-scores were drawn using Heatmapper 50.

Analysis of KEGG pathways 51 was performed using the gseapy package 52, 53. Normalized enrichment scores (nes) were reported for the top-10% regulated pathways as well as for 39 pre-selected pathways involved in cancer and in the sensitivity to ionizing radiation 54. Using an explorative approach, positive and negative enrichment in pathways was considered significant at false discovery rates (fdr) < 0.25. A specific subset of these enrichment pathways was extracted representing the additional effects of ET on the regular response to PRRT and lists of up-regulated leading-edge genes were further analyzed using the 'Protein Analysis Through Evolutionary Relationships' (PANTHER) classification system 55, 56. Protein classes encoded by up-regulated leading edge genes were visualized using Meta-Chart (www.meta-chart-com). Further details on pre-selection of pathways and on extraction of enrichment gene sets are provided in *Supplemental Information 1.7.*

### Real-time RT-PCR

Preparation of cDNA from mouse RNA and amplification using gene-specific primers are described in *Supplemental Information 1.8*.

### Statistical analysis

Statistical analysis was performed using Prism 8 (GraphPad Software, San Diego CA, USA). Data are presented as means ± SEM and *n* represents the number of data sets investigated. Significance of differences was tested using ANOVA and Sidak *post-hoc* test. Significance of relationships was tested using Spearman's linear correlation test and displayed as correlation coefficient (*r*_s_). Differences were considered significant at *P-*values < 0.05. Responder thresholds were calculated from the mean value of a reference cohort + two times typical error (2×TE).

## Results

### Tolerated doses of VPA and DAC

**Figure 1:**
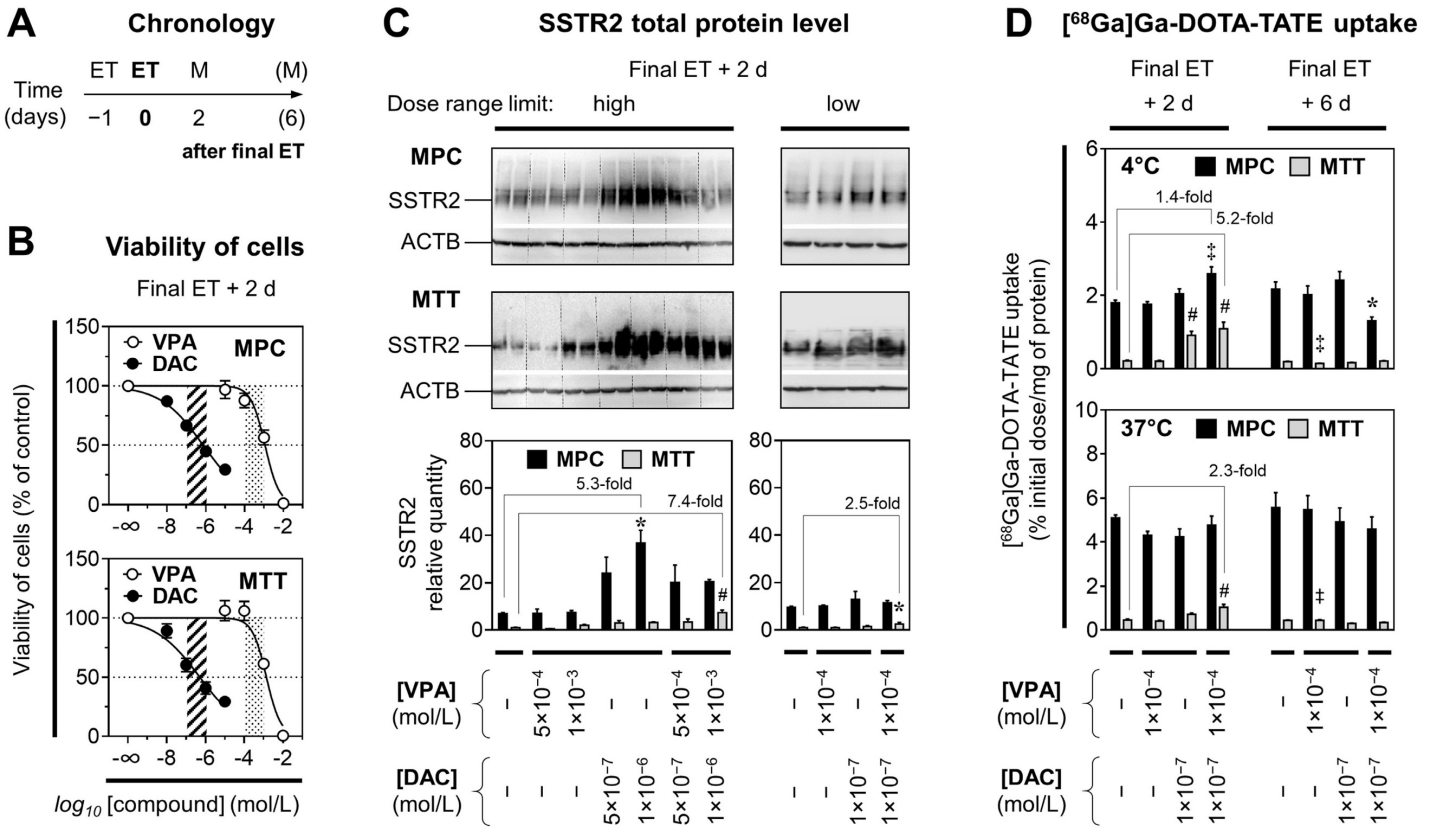
** Viability, SSTR2 protein levels, and [^68^Ga]Ga-DOTA-TATE uptake of MPC and MTT cells treated with epigenetic drugs; (A)** Sequence of investigations after treatment with VPA and DAC as single and combination doses with different concentrations; (M) measurements; **(B)** Dose-response-curves showing reduction of cell viability in response to increasing concentrations of epigenetic drugs; dose ranges of DAC (dashed area) and VPA (dotted area) for investigations on SSTR2 modulation: **(C)** Western-Blot analysis showing changes in SSTR2 protein levels of cells upon ET; relative quantity: SSTR2/ACTB ratio normalized to the average of MTT [Controls]; **(D)** SSTR2 radioligand assay showing changes in [^68^Ga]Ga-DOTA-TATE uptake of cells upon ET; significance of differences: * *P* < 0.05, ‡ *P* < 0.01, # *P* < 0.001.

To investigate whether VPA and DAC stimulate SSTR2 in PCCs/PGLs, we considered effects of epigenetic modulation to be of primary importance rather than cytotoxic effects. Hence, appropriate doses for both compounds were estimated based on cell viability studies (Figure 1A). The cytotoxic effects of ET were similar in MPC and MTT cells showing LD_50_ values of 1.3×10^-3^ mol/L for VPA and 6.3×10^-7^ mol/L for DAC (Figure 1B). For both cell lines, hill slopes of the VPA dose-response curves (-1.2 ± 0.3) were considerably steeper compared to DAC (-0.4 ± 0.02) indicating that the effective dose range of VPA is smaller compared to DAC. The tolerated dose for modulating SSTR2 was defined as concentrations between LD_50_‒10% and LD_50_ (10^‒4^ to 10^-3^ mol/L for VPA and 10^-7^ to 10^-6^ mol/L for DAC).

### SSTR2 protein levels in response to epigenetic drugs *in vitro*

Densitometric analyses of protein bands (SSTR2/ACTB ratios) showed that SSTR2 levels in MPC [Control] cells were between 7- and 10-fold higher compared to MTT [Control] cells (Figure 1C). Treatment of MPC and MTT cells with VPA and DAC showed concentration- and combination-dependent effects on SSTR2 levels. MPC cells exhibited the strongest response when treated with DAC at 10^-6^ mol/L, resulting in a 5.3-fold up-regulation of SSTR2 compared to controls. MTT cells exhibited the strongest response when treated with a combination of VPA at 10^-3^ mol/L and DAC at 10^-6^ mol/L resulting in a 7.4-fold up-regulation of SSTR2 compared to controls. These effects were smaller at lower epigenetic drug concentrations.

### [^68^Ga]Ga-DOTA-TATE uptake in response to epigenetic drugs *in vitro*

Functional investigations on SSTR2 using [^68^Ga]Ga-DOTA-TATE showed that radioligand uptake was about 10-fold higher in MPC cells compared to MTT cells (Figure 1D). Treatments of MPC cells with VPA (10^-3^ mol/L) and DAC (5×10^-7^ mol/L) resulted in reduced radiotracer uptake at 37 °C, a condition where SSTR2-mediated endocytosis and intracellular trafficking occurs upon ligand binding (see *Supplemental Information 2.1* for details). This reduction was attributed to cytotoxic effects. Hence the following experiments were performed with concentrations at the lower limits of the defined dose ranges for VPA (10^-4^ mol/L) and DAC (10^-7^ mol/L).

At 4 °C, a condition where SSTR2-mediated endocytosis and intracellular trafficking is largely impaired, MPC cells exhibited the strongest stimulation in radioligand binding when treated with a combination of VPA and DAC that resulted in a significant 1.4-fold increase two days after final ET compared to controls (Figure 1D). The same treatment did not show effects at 37 °C.

MTT cells exhibited the strongest stimulation in radioligand binding when treated with the combination of VPA and DAC. A significant 5.2-fold increase compared to controls was detected at 4 °C, the same treatment showed a 2.3-fold increase at 37 °C. Up-regulation of [^68^Ga]Ga-DOTA-TATE uptake in MPC and MTT cells by VPA and DAC was short-lived and not maintained much beyond epigenetic drug application, since six days after ET all effects were gone.

### [^64^Cu]Cu-DOTA-TATE binding constants in response to epigenetic drugs *in vitro*

SSTR2 saturation assays with cell homogenates showed that ET with a combination of VPA and DAC increased the specific binding capacity (*B*_max_) for [^64^Cu]Cu-DOTA-TATE in both MPC and MTT cells, respectively (Table 2, *Supplemental Figure S1B*). Without treatment, the *B*_max_ value of MPC cells was 8-fold higher compared to MTT cells. Upon ET, *B*_max_ values significantly increased 1.7-fold in MPC cells and 3.3-fold in MTT cells compared to controls. ET had no effect on binding affinities (*K*_d_).

### [^64^Cu]Cu-DOTA-TATE uptake of allograft tumors in response to epigenetic drugs

**Figure 2:**
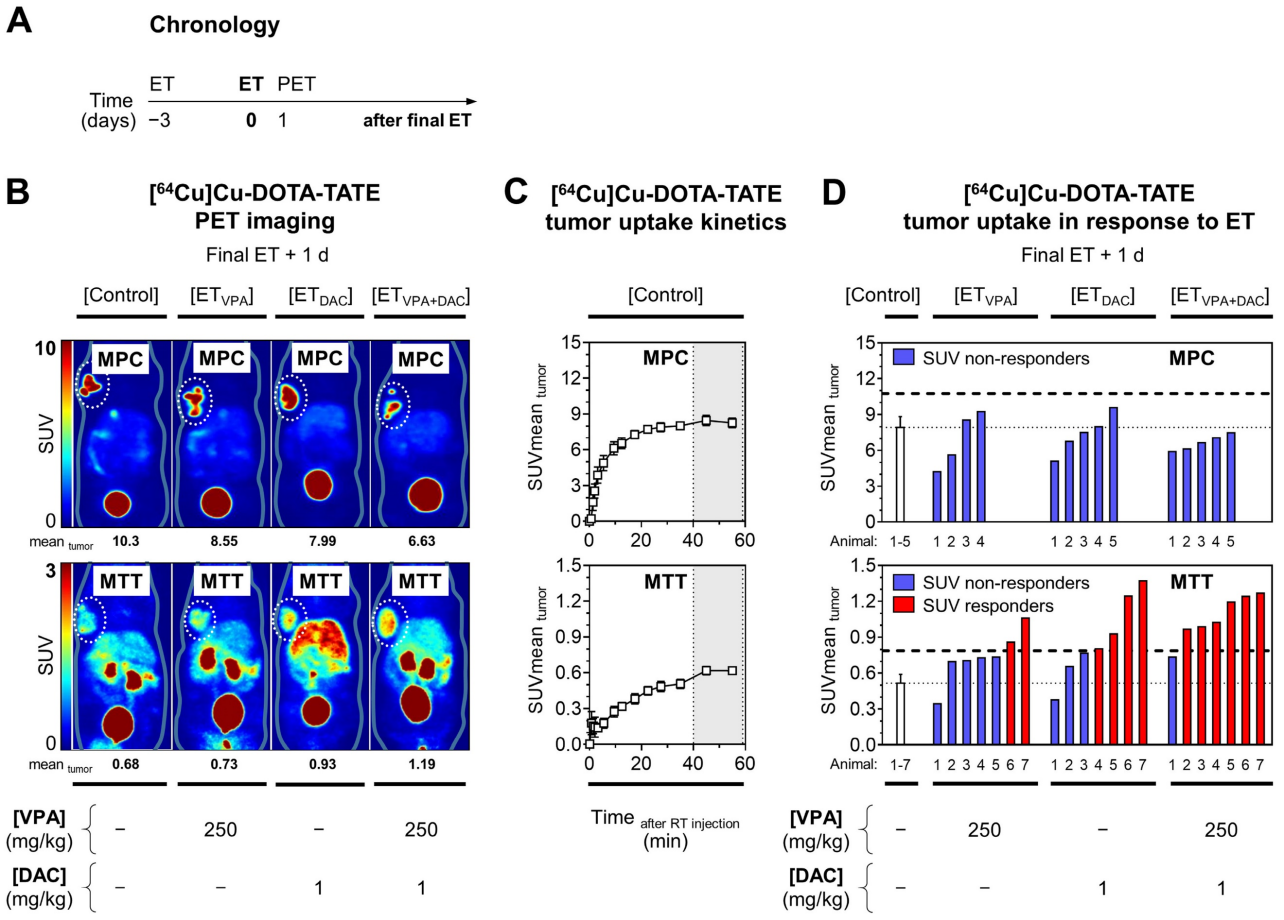
** PET imaging with [^64^Cu]Cu-DOTA-TATE in MPC and MTT allograft mice treated with epigenetic drugs. (A)** Sequence of investigations after treatment with VPA (250 mg/kg) and DAC (1 mg/kg) as single and combination doses, respectively followed by PET imaging with [^64^Cu]Cu-DOTA-TATE (10 MBq/animal, equivalent to 0.25 nmol); **(B)** Maximum intensity projections of PET images presented at different SUV color scaling: MPC (0-10); MTT (0-3); (dotted regions) uptake in tumors in response to ET; **(C)** Kinetic profiles of radiotracer uptake in untreated tumors measured within 60 min after injection; dotted vertical lines indicate the time frame (40‒60 min) further analyzed for comparing ET effects; **(D)** Changes in radiotracer uptake of tumors in response to ET; (dashed horizontal lines) SUV responder thresholds compared to [Control] cohorts; (RT) radiotracer; see *Supplemental Information 2.2* for additional data.

Effects of ET on [^64^Cu]Cu-DOTA-TATE uptake in tumors were investigated *in vivo* in MPC and MTT allograft mice (Figure 2A). Small-animal PET imaging showed that effects of ET on radiotracer uptake in tumors varied depending on the tumor type and the mode of treatment (Figure 2B).

Without treatment, kinetic profiles of [^64^Cu]Cu-DOTA-TATE uptake in tumors showed that standardized uptake values (region-averaged SUVmean) reached a plateau between 40 and 60 min after intravenous injection that was 14-fold higher in MPC allografts (8.26) compared to MTT allografts (0.62) (Figure 2C).

The already high [^64^Cu]Cu-DOTA-TATE uptake in MPC tumors was reduced by ET, most likely due to cytotoxic effects of the treatment **(**Figure 2D**,** see *Supplemental Information 2.2* for details). MTT tumors showed significant increases in [^64^Cu]Cu-DOTA-TATE uptake, although uptake levels were considerably lower compared to MPC tumors. The average SUVmean of MTT tumors in the control cohort increased 1.4-fold with VPA, 1.7-fold with DAC (*P* < 0.05), and 2.1-fold with the combination (*P* < 0.01). The percentage of MTT responders was 29% (2/7), 57% (4/7), and 86% (6/7) for VPA, DAC, and the combination, respectively. Correlation analyses showed that changes in [^64^Cu]Cu-DOTA-TATE uptake by tumors were independent from tumor volume and from individual differences in radiotracer retention in blood, liver, and kidneys (see *Supplemental Figures S4 and S5* for details).

### [^177^Lu]Lu-DOTA-TATE uptake and growth of allograft tumors in response to epigenetic drugs

**Figure 3:**
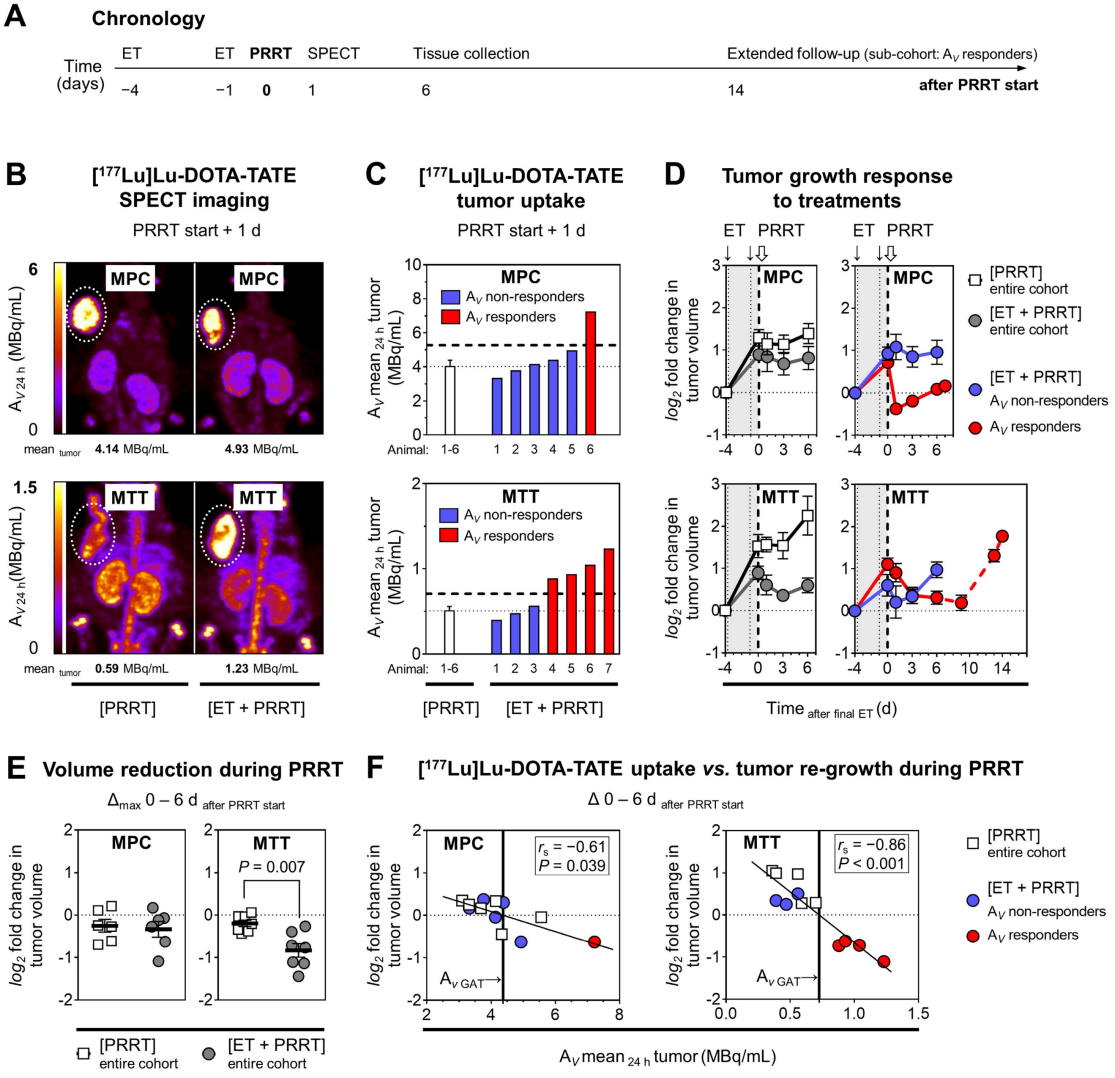
** PRRT with [^177^Lu]Lu-DOTA-TATE in MPC and MTT allograft mice treated with epigenetic drugs; (A)** Sequence of investigations after ET with VPA (250 mg/kg) and DAC (1 mg/kg) as combination doses followed by PRRT with [^177^Lu]Lu-DOTA-TATE (70 MBq/animal, equivalent to 1.2 nmol), **(B)** Maximum intensity projections of SPECT images; (A*_V_*_24 h_) activity concentration 24 hours after radiotracer injection; (dotted regions) uptake in tumors; **(C)** Effects of ET on activity concentrations in tumors; (dashed horizontal lines) A*_V_* responder thresholds compared to [PRRT] cohorts; **(D)** Changes in tumor volume in response to treatments; (*log_2_* fold changes) number of volume doublings compared to treatment start; (dotted vertical lines) time points of ET; (dashed vertical lines) initiation of PRRT; (dashed red connecting curve) extended follow-up in a sub-cohort of MTT allograft mice with highest activity concentrations in tumors (*n* = 3); **(E)** Maximum changes in tumor volume during six days of PRRT; **(F)** Correlation between the initial activity concentrations in tumors and tumor re-growth; (GAT) growth arrest of tumors; see *Supplemental Information 2.3* for additional data.

The effects of ET on the efficacy of peptide receptor radionuclide therapy (PRRT) with [^177^Lu]Lu-DOTA-TATE were investigated in MPC and MTT allograft mice (Figure 3A). The average activity concentration resulting from [^177^Lu]Lu-DOTA-TATE uptake in tumors (A*_V_* mean_ 24h_) was 8-fold higher in MPC allograft mice (4.02 MBq/mL) compared to MTT allograft mice (0.50 MBq/mL) (Figure 3B-C). This was consistent with other readouts from *in vitro* experiments and PET imaging. The activity concentration of [^177^Lu]Lu-DOTA-TATE increased slightly upon ET by 1.2-fold to 4.62 MBq/mL in MPC allografts. Only 17% (1/6) of these mice were classified as treatment responders. In MTT allografts, the average activity concentration of [^177^Lu]Lu-DOTA-TATE increased upon ET by 1.6-fold to 0.79 MBq/mL (*P* < 0.05), and 57% (4/7) of the mice responded to the treatment (see *Supplemental Information 2.3* for further details).

The combination of ET and PRRT had similar growth-reducing effects as PRRT monotherapy (18% *versus* 14% volume reduction; 10 *versus* 9 days growth delay) in MPC tumors (Figure 3D). In MTT tumors, ET together with PRRT significantly decreased tumor growth compared to PRRT alone (46% *versus* 14% volume reduction, 13 *versus* 3 days growth delay) (Figure 3E).

Linear relationships between the activity concentration of [^177^Lu]Lu-DOTA-TATE and the reduction of tumor growth confirmed that the additional effect of ET can be attributed to increased radiotracer uptake (Figure 3F). A 6-fold higher activity concentration was required in MPC compared to MTT tumors to achieve similar growth-reducing effects. This indicates that MPC tumors are more radioresistant. All tumors included in subsequent transcriptional analyses showed re-growth at the time of tissue preservation (six days after PRRT start).

### Status of Sstr2/SSTR2 in allograft tumors in response to epigenetic drugs and [^177^Lu]Lu-DOTA-TATE

To further characterize molecular effects of ET treatment in combination with PRRT, DNA, mRNA, and protein samples were obtained from vehicle-treated [Control] and [ET] cohorts, as well as from [PRRT] and [ET + PRRT] sub-cohorts, each representing the entire treatment cohort by a similar mean activity level in tumors (*Supplemental Table S5*).

**Figure 4:**
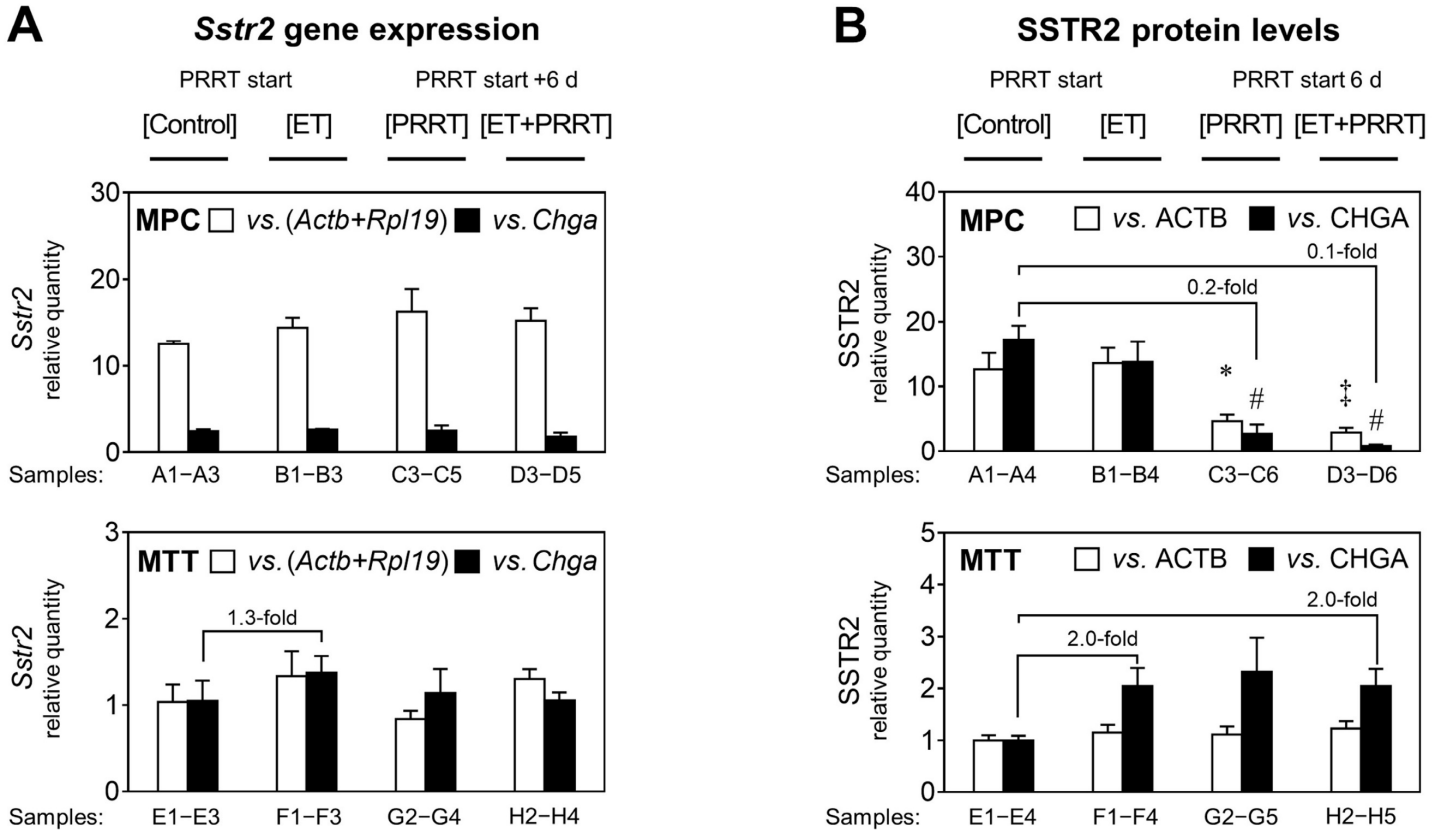
***Sstr2* expression and SSTR2 protein in MPC and MTT tumors treated with epigenetic drugs and PRRT**; reported for treatment sub-cohorts; ET: treatment with VPA (250 mg/kg ) and DAC (1 mg/kg) as combination doses on days -4 and -1; PRRT: treatment with [^177^Lu]Lu-DOTA-TATE (70 MBq/animal, equivalent to 1.2 nmol) as a single dose on day 0; **(A)** *Sstr2* gene expression measured by real-time RT-PCR using (*Actb+Rpl19*) and *Chga* as reference genes; relative quantity: ΔΔCt values normalized to the average of MTT [Controls]; **(B)** SSTR2 protein measured through densitometric analysis of immunoblots using ACTB and CHGA as reference proteins; relative quantity: band intensity ratios normalized to the average of MTT [Controls]; *Actb+Rpl19* and ACTB were used to compare levels between the different cell lines, whereas *Chga*/CHGA is a marker for pheochromocytes and treatment effects for each cell line are better attributable to the fraction of pheochromocytes within the tumor (see also *Supplemental Figures S 8 and S 9*); significance of differences: * *P* < 0.05; ‡ *P* < 0.01, # *P* < 0.001.

RT-PCR confirmed that MPC tumors had significantly higher *Sstr2* levels than MTT, with a difference of 12-fold (Figure 4A). Since *Chga* expression differs between MPC and MTT cells in monolayer culture (data not shown), this reference gene cannot be used for comparison between cell lines, instead it is a specific marker for pheochromocytes within the allograft and is used for comparing the different treatments within each tumor group. *Sstr2* gene expression in both MPC and MTT tumors was largely unaffected by ET. MTT cells had slightly increased *Sstr2* levels upon [ET]; however, statistical significance was not reached. SSTR2 protein levels reflected the differences in gene expression between the cell lines (Figure 4B). MPC allografts showed 13-fold higher SSTR2 (relative to ACTB) than MTT tumors. The MPC cell fraction in tumors showed reduced SSTR2 (relative to CHGA) by 0.2 and 0.1-fold in response to [PRRT] and [ET + PRRT] treatments, respectively. The MTT cell fraction in tumors had slightly increased SSTR2 levels in response to treatments.

Since it has been proposed that *Sstr2* expression is regulated by DNA methylation of a CpG island within the *Sstr2* promoter, we tested whether promoter methylation was implicated in the observed changes. Both MPC and MTT tumors as well as the corresponding monolayer cell cultures did not show methylation of the *Sstr2* promoter (see *Supplemental Information 2.4* for additional data).

### Gene expression signature of allograft tumors in response to epigenetic drugs and [^177^Lu]Lu‑DOTA-TATE

Transcriptional changes in tumors between [ET] and [Control] were not as pronounced as changes induced by [PRRT]. Gene set enrichment analysis (GSEA) identified similar differentially regulated pathways by [ET] (compared to [Control]) in MPC and MTT tumors, however, in different direction; e.g., 'cholesterol metabolism' and 'DNA replication' were down-regulated and 'steroid biosynthesis' and 'central carbon metabolism' were up-regulated in MPC, but the reverse was true in MTT. The top-regulated gene sets in MPC and MTT tumors showed a number of pathways attributed to treatment-associated tissue damage and infiltration of leukocytes, e.g., 'complement and coagulation cascades', 'leukocyte transendothelial migration', 'cytokine and cytokine receptor interaction, and 'phagosome'. Therefore, a more specific pathway analysis was performed focusing on 39 pre-selected gene sets known to be involved in cancer and treatment resistance (see *Supplemental Information 2.5 and 2.6* for details).

**Figure 5:**
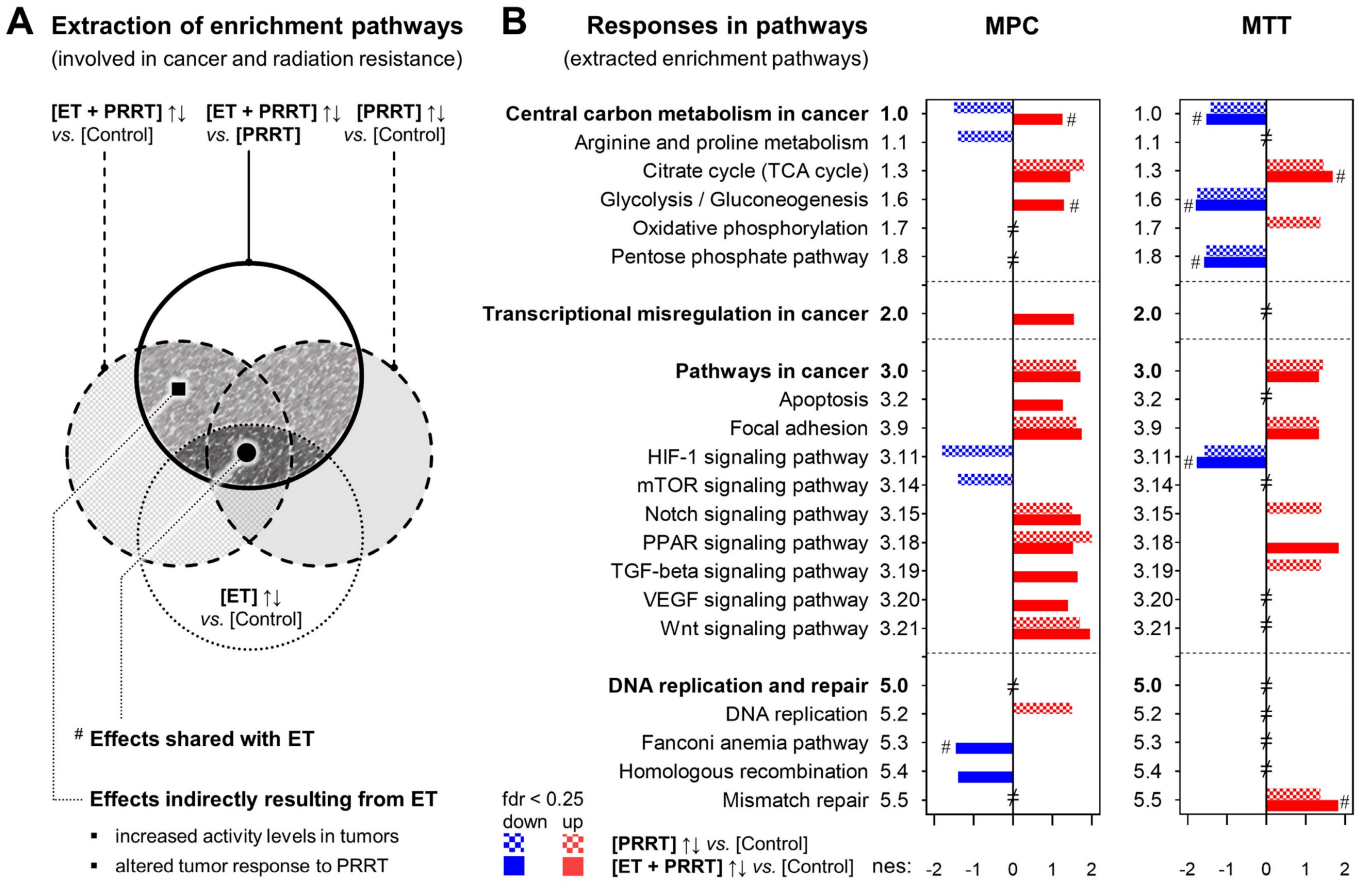
** Additional effects of ET on the transcriptional response of MPC and MTT tumors to PRRT;** reported for treatment sub-cohorts (*n* = 3); ET: treatment with VPA (250 mg/kg ) and DAC (1 mg/kg) as combination doses on days -4 and -1; PRRT: treatment with [^177^Lu]Lu-DOTA-TATE (70 MBq/animal, equivalent to 1.2 nmol) as a single dose on day 0; **(A)** Extraction of KEGG pathways representing the additional effects of ET on the regular response to PRRT; (full hatched area) extracted enrichment pathways included in further analyses; (fdr < 0.25) false-detection rate threshold for positive and negative pathway enrichment; **(B)** KEGG pathways (from A) listed according to differential responses of [PRRT] and [ET + PRRT] compared to [Control]; (nes) normalized enrichment score; (#) response shared with initial [ET]; (≠) not significantly altered under any treatment condition; additional information on pathway selection can be found in *Supplemental Information 1.7 and 2.6*.

Analysis of pre-selected gene sets identified 18 pathways in MPC tumors and 12 pathways in MTT tumors, each showing a significantly different transcriptional response to [ET + PRRT] compared to [PRRT] (Figure 5A and B). Furthermore, leading-edge analysis identified 68 genes in MPC tumors and 92 genes in MTT tumors that were differentially expressed at least in one of the three treatment groups compared to [Control] (Tables 3 and 4). Notably, most of the pathway effects shared between [ET + PRRT] and the initial [ET] were not related to the same leading-edge genes.

Compared to the response of MPC tumors to [PRRT] monotherapy, [ET + PRRT] combination therapy most prominently induced positive enrichment in 'cancer central carbon metabolism', 'glycolysis', 'transcriptional misregulation in cancer', 'apoptosis', 'TGF-beta signaling', and 'VEGF signaling', and caused negative enrichment in the 'Fanconi anemia pathway' and in 'homologous recombination' (Table 3). In MTT tumors, [ET + PRRT] combination therapy in comparison to [PRRT] monotherapy most prominently induced positive enrichment in 'PPAR signaling', attenuated positive enrichment in 'oxidative phosphorylation', 'Notch signaling', and 'TGF-beta signaling' (Table 4).

Transcriptional responses of MPC and MTT tumors in other pathways were less pronounced but still detectable in leading-edge gene analysis. Besides the leading-edge genes involved in the extracted enrichment pathways, [^177^Lu]Lu-DOTA-TATE treatment also induced, amongst others, transcriptional upregulation of *Tnxb* and *Tnc* genes encoding different members of the structure-supporting tenascin glycoprotein family (see *Supplemental Information 2.6* for additional data).

## Discussion

This study demonstrates that the combination of VPA and DAC is capable of increasing SSTR2 protein levels along with radiotracer uptake *in vitro* in two PCC/PGL models, one with high (MPC) and an another with low (MTT) SSTR2 levels. Drug effects were less pronounced in *in vivo* experiments, where predominantly MTT tumors responded to epigenetic treatment with significantly elevated SSTR2 radioligand uptake. In both models, complementary effects of epigenetic drugs and [^177^Lu]Lu-DOTA-TATE were associated with additional reduction in tumor growth and specific transcriptional responses in gene sets involved in cancer and treatment resistance.

VPA is a clinically approved and widely used anticonvulsant and mood stabilizer 57. Additionally, VPA is known to be a potent HDAC inhibitor affecting DNA methylation and antitumor activity 58, 59. Deacetylation is generally linked to reduced gene expression and accordingly, HDAC inhibitors lead to accumulation of acetylated histones, an open chromatin conformation, and subsequently to enhanced transcription of genes 60, 61. In contrast to other HDAC inhibitors, the advantages of VPA are its oral bioavailability, a good safety profile and tolerability with long experience of use 57, 62. VPA was shown to stimulate SSTR2 in neuroendocrine tumor models, but also induces apoptosis and cell cycle arrest 23. There is a growing body of evidence that HDAC inhibitors, when given either pre- or post-radiation therapy, can provide a synergistic radiosensitization response 63.

DAC (also known as decitabine®) is a cytostatic agent with approval as an orphan drug for the treatment of myelodysplastic syndrome and chronic myelomonocytic leukemia in the US 64 and as 2^nd^ line treatment for acute myeloid leukemia in the EU 65. As a deoxynucleoside analog (“antimetabolite“), DAC is incorporated into the DNA instead of cytosine and inhibits DNMT by covalent addition and enzyme trapping, resulting in the depletion of the enzyme, demethylation of replicating DNA, and apoptosis of tumor cells 60, 61. DNA demethylation is associated with increased transcription of specific genes 66, 67.

SSTR2-inducing treatments are especially important for neuroendocrine tumor patients not eligible for targeted therapies due to insufficient or undetectable SSTR2 levels 16. About 28% of PCCs and 13% of PGLs show low or undetectable SSTR2 9. In our models, induction of SSTR2 protein content was most efficient in the LD_50_ dose range, whereas stimulation of [^68^Ga]Ga-/[^64^Cu]Cu-DOTA-TATE binding was highest at less toxic doses of the epigenetic drugs. It is possible that western blot analysis does not sufficiently resolve the moderate changes in SSTR2 protein, in particular in MTT cell lysates. Strong membrane incorporation of G-Protein-coupled receptors may affect protein extraction, migration characteristics, and immunodetection to an extent that may override the regulatory effects by epigenetic drugs. For this reason, lower doses were investigated using radioligand assays, which have much higher sensitivity compared to immunoblotting.

Consistent with radioligand uptake *in vitro* a more pronounced anti-tumor effect *in vivo* was associated with attenuated [^64^Cu]Cu-DOTA-TATE uptake in MPC allograft mice, indicating that cytotoxic effects of the epigenetic drugs outweigh stimulatory effects on SSTR2 content. We speculate that energy-dependent cellular processes required, e.g., for maintenance of short-term SSTR2 recycling following ligand-induced endocytosis might be impaired by higher drug concentrations. Therefore, only a very narrow dose range allowed for increasing both SSTR2 content and uptake of radioligands at the same time.

SSTR2-inducing effects of DMNT and HDAC inhibitors are known to vary among different tumor models 17. Previous results suggest that especially neuroendocrine tumor cell lines with low (i.e. BON-1) or intermediate (i.e. NCI-H727 and TT cells) SSTR2 levels are susceptible to epigenetic treatment, whereas upregulation in neuroendocrine tumor cell lines with high SSTR2 levels is less effective (i.e. GOT-1 and MZ-CRC-1 cells) 16, 20. Consistent with these findings, the relative increase in SSTR2 protein and radiotracer uptake was more pronounced in MTT compared to MPC cells.

The stimulatory effect of epigenetic drugs on [^64^Cu]Cu-DOTA-TATE uptake in MTT tumors was comparable to what was reported for BON‑1 xenografts in mice, where the uptake of [^68^Ga]Ga-DOTA-TATE increased approximately 2-fold after treatment with DAC 17. On the other hand, a recent pilot imaging study in patients with midgut neuroendocrine liver metastases showed only an increase of 10% in [^68^Ga]Ga-DOTA-TATE uptake in tumors after a short course of vorinostat 68. This HDAC inhibitor, however, is not the most efficacious epigenetic drug to induce [^68^Ga]Ga-DOTA-TATE or [^68^Ga]Ga-DOTA-TOC uptake 17, 25. Short‐term epigenetic treatment with VPA and the DNMT inhibitor hydralazine had no stimulating effect on [^68^Ga]Ga‐DOTA-TATE uptake in nine patients with well‐differentiated neuroendocrine tumors with low baseline expression of SSTRs 69. These studies demonstrate that further investigations are required to fully appreciate and optimize the clinical potential for epigenetic drugs in improving PRRT.

HDAC and DNMT inhibitors can have different impacts depending on the cellular background 17. For example, in QGP-1 cells, DAC induced, but VPA inhibited *SSTR2* gene expression. In combined application, VPA attenuated the stimulating effect of DAC. On the other hand, in BON-1 cells, *SSTR2* expression increased in response to both VPA and DAC monotherapies, and combined application even showed synergistic effects 18. In MPC and MTT models, DAC induced SSTR2 protein levels and radiotracer uptake more effectively compared to VPA, and although combined application of the drugs was slightly more effective compared to monotherapies, no synergistic effect occurred. In line with our findings, it was shown that the stimulating effects of VPA on *SSTR2* expression and [^111^In]In-DOTA-TATE uptake in neuroendocrine tumor cell lines were largely reversible one day after VPA withdrawal 26. In MPC and MTT cells, effects persisted for two days after treatment and were completely reversed within six days. This suggests that, also for PCC/PGL, proper timing of epigenetic treatments is an important factor.

Additionally, treatment doses and schedule might require further optimization. We chose short-term VPA and DAC treatments at fixed and well-tolerated doses, which were reported to effectively induce epigenetic changes in mice 41-43. The estimated human equivalent doses corresponded to 20‒30% of the clinically applied tumor-suppressive doses in patients 44, 45. Lower doses were used, since we focused on the epigenetic modulation effects rather than the cytotoxic effects of VPA and DAC. Our results showed that SSTR2 induction and [^64^Cu]Cu-DOTA-TATE binding occurred to a lesser extent in tumor-bearing mice compared to cell cultures. For the intraperitoneal doses applied in this study, serum levels of the epigenetic drugs have been reported to normalize within eight hours for VPA 43 and within two hours for DAC 70, suggesting that shorter treatment intervals may yield better results. It needs to be determined, whether such a strategy benefits epigenetic modulation or whether it increases cytotoxic effects to the point that they outweigh the stimulatory effects on SSTR2 and radiotracer uptake.

PET imaging of tumor allografts using [^64^Cu]Cu-DOTA-TATE had the disadvantage of high liver background, especially at low-intensity scaling for visualizing the low radiotracer uptake in MTT tumors. Due to the well-defined subcutaneous location of the tumors, this limitation did not affect image analysis of radiotracer uptake in these particular regions. [^64^Cu]Cu-DOTA-TATE was shown to have excellent performance in patients and its routine use for PET imaging of neuroendocrine tumors was proposed 71.

In contrast to a recent report by Klomp *et al.* on SSTR2 modulating effects of VPA in NCI-H69 xenograft mice 72, we did not detect large differences in radiotracer biodistribution in response to epigenetic drugs, nor did we detect a relationship between radiotracer uptake in tumors and the retention in blood, liver, and kidneys. Compared to our study, in which PET imaging was performed 24 hours after the final epigenetic treatment, Klomp *et al*. performed PET imaging as early as four hours after treatment, which most likely explains the different observations.

In agreement with our [^64^Cu]Cu-DOTA-TATE data, the combination of VPA and DAC also stimulated the uptake of [^177^Lu]Lu-DOTA-TATE in MTT tumors. On the other hand, uptake in MPC tumors remained largely unchanged upon treatment with epigenetic modifiers. Activity concentrations of [^177^Lu]Lu-DOTA-TATE were measured in tumors approximately 24 hours after initiation of PRRT *via* quantitative SPECT imaging and were correlated with treatment effects. Although SPECT scans for [^177^Lu]Lu-DOTA-TATE dosimetry in patients are often initiated 24 hours after PRRT start, dose estimation requires multiple scans at different time points. The actual β^-^ radiation dose (^177^Lu) absorbed by tumors over time would have been a more desirable reference value, but the activity concentration represents the uptake fraction of the radionuclide drug and is therefore an acceptable reference value that largely determines the absorbed dose. Although epigenetic drugs had a stimulatory effect on [^177^Lu]Lu-DOTA-TATE uptake in most MTT tumors, non-responders to the treatment occurred, indicating that the individual efficacy depends on various experimental and animal-specific conditions, which might include biodistribution and pharmacokinetics of the epigenetic drug after intraperitoneal injection, heterogeneity in vascularization and perfusion of tumors, heterogeneity in basal SSTR2 content of tumors, and drug-related toxicity attenuating radiotracer uptake in tumor cells.

Our study furthermore demonstrates that the SSTR2 stimulating effects of epigenetic treatments, in particular those of DAC, were unrelated to *Sstr2* promoter methylation, since the promoter was found to be unmethylated in all MPC and MTT models. This is in agreement with a study in neuroendocrine cell lines, which identified acetylation at lysine-9 of histone 3 to be potentially involved in regulating *SSTR2* expression 18. HDAC inhibitors were also shown to affect lipid homeostasis that can in turn influence the stability of G-protein coupled receptors 73, 74. Treatment of MPC and MTT tumors with epigenetic drugs altered the expression of components of the cholesterol synthesis pathway and could therefore lead to changes in the membrane composition. Further investigations are needed to fully understand the mechanism of action, but are beyond the scope of the present report.

MPC and MTT tumors showed largely inverse responses to epigenetic drugs as determined four days after treatment start. This is reflected in the different sensitivity of the models to the combination of epigenetic drugs with PRRT. MPC tumors continued to grow largely unaffected by the combination treatment, similar to the PRRT monotherapy group, leading to increasing restrictions in oxygen supply, hypoxia signaling, and a central carbon metabolism that is more dependent on glycolysis. In contrast, the faster growing but also more treatment sensitive MTT tumors were substantially attenuated by the combination of epigenetic drugs and PRRT, resulting in increased cell death associated with re-oxygenation and a central carbon metabolism that is less dependent on glycolysis. Additionally, the transcriptional up-regulation in pathways involved in p53 signaling and DNA damage repair are also consistent with the stronger impact of the epigenetic drugs on MTT tumors.

Specific transcriptional responses of tumors to the combination of epigenetic treatment and PRRT can be attributed to two separate effects; first, to direct effects of the epigenetic drugs that occurred immediately after pre-treatment and persisted throughout the PRRT phase, and second, to indirect effects of the epigenetic drugs that occurred first during PRRT as a result of increased [^177^Lu]Lu-DOTA-TATE uptake. Since a recent study showed that epigenetic treatment with VPA and DAC did not sensitize HEK293 and PC3 cells to X-ray irradiation *in vitro* 27, we suspect the observed additional treatment effects to result from increased [^177^Lu]Lu-DOTA-TATE uptake and higher radiation doses absorbed by the tumors. On the other hand, there is literature supporting the radiosensitizing effects of HDAC inhibitors in neuroendocrine tumor cell lines 19, 26. Further investigations on these effects in pheochromocytoma allograft mice would require co-treatment with epigenetic drugs during the PRRT phase.

In MPC tumors, additional transcriptional responses to PRRT induced by epigenetic treatment indicate both complementary and protective effects at the same time. Complementary effects include down-regulated gene expression in 'Fanconi anemia pathway' and 'homologous recombination'. The Fanconi anemia pathway is activated primarily during S phase for removal of critical DNA interstrand crosslinks 75. Homologous recombination is one of the major pathways for repair of DNA double strand breaks 76. Protective effects in MPC tumors include upregulated gene expression in 'glycolysis', 'TGF-β signaling', and 'VEGF signaling'. A glycolysis-dependent metabolic state, even under aerobic conditions, is often associated with treatment resistance in different cancers 77. TGF-β has tumor-promoting effects in established tumors and plays a critical role in cancer radiotherapy inducing epithelial-mesenchymal transition, cancer stem cells and cancer-associated fibroblast, and suppresses the immune system 78. VEGF is an important pro-angiogenic factor that promotes tumor angiogenesis and protects tumor vessels from radiation-related damage contributing to tumor cell re-oxygenation accompanied by increased DNA replication and radioresistance 79, 80. In MTT tumors, higher sensitivity to PRRT upon combination with epigenetic treatment was associated with increased gene expression in 'PPAR signaling', which sensitizes cancer cells to ionizing radiation 81, 82. Interference with any of the six above mentioned pathways might modulate sensitivity of PCC/PGLs against PRRT.

On single gene-level, transcriptional responses of MPC and MTT tumors to [^177^Lu]Lu-DOTA-TATE showed similarities with a report on susceptible genes and molecular pathways related to heavy ion irradiation in oral squamous cell carcinoma cells 83. On the other hand, a study on [^177^Lu]Lu-DOTA-TATE treatment of human small intestine neuroendocrine GOT1 tumors in nude mice described a largely different transcriptional response, except for the shared involvement of *Tgfb*, *Ngfr* and *Id1* 84. Up-regulation of *Tnxb* and *Tnc* genes in MPC and MTT tumors upon [^177^Lu]Lu-DOTA-TATE treatment indicate that tenascin glycoproteins may be involved in tumor recurrence. In PCC/PGL, increased tenascin-X has been reported to be associated with metastatic disease 85. Tenascin-C and VEGF-A produced by S100A4-positive stromal cells have been reported to contribute to metastatic colonialization 86.

## Conclusion

Mouse pheochromocytoma cell lines are responsive to SSTR2-inducing epigenetic treatments; however, successful SSTR2 stimulation *in vitro* does not necessarily predict the efficacy of the epigenetic drugs in the corresponding allograft models. Further investigations are required to identify the molecular determinants for the varying susceptibility of PCC/PGL cells to SSTR2-inducing effects. Epigenetic modifiers have potential for clinical application in combination with PRRT, specifically in tumors with low SSTR2 levels. Treatment schedule and dose of the epigenetic drugs require thorough evaluation in the clinical setting to find optimal conditions for SSTR2 induction and treatment efficacy. PET imaging, e.g., with [^68^Ga]Ga-DOTA-TATE after first epigenetic drug exposure could be a useful tool to identify patients responding to SSTR2 induction and benefitting from such an approach.

## Supplementary Material

Supplementary methods, figures and tables.

## Figures and Tables

**Table 1:** Animal cohorts for investigating the effects of ET on SSTR2-targeted PRRT imaging and treatment

Cohort label	ET	Radiopharmaceutical	Animals *n* (experiments)		Tumor samples^1^	
			MPC	MTT	MPC	MTT
**PET imaging and biodistribution**						
[Control]	Vehicle (PBS)	[^64^Cu]Cu-DOTA-TATE	5 (2, 2, 1)	7 (1, 2, 2, 2)	-	-
[ET _VPA_]	VPA	[^64^Cu]Cu-DOTA-TATE	4 (1, 2, 1)	7 (1, 2, 2, 2)	-	-
[ET _DAC_]	DAC	[^64^Cu]Cu-DOTA-TATE	5 (2, 2, 1)	7 (1, 2, 2, 2)	-	-
[ET _VPA + DAC_]	VPA + DAC	[^64^Cu]Cu-DOTA-TATE	5 (2, 2, 1)	7 (1, 2, 2, 2)	-	-
**PRRT, SPECT imaging, RNAseq, and Western-Blot**						
[Control]	Vehicle (PBS)	w/o	3	3	A	E
[ET]	VPA + DAC	w/o	3	3	B	F
[PRRT]	Vehicle (PBS)	[^177^Lu]Lu-DOTA-TATE	6 (3, 3)	7 (4, 2, 1)	C	G
[ET + PRRT]	VPA + DAC	[^177^Lu]Lu-DOTA-TATE	6 (3, 3)	7 (4, 2, 1)	D	H

Animal numbers in parentheses represent animal numbers included in independent experiments; (ET) epigenetic treatment; (PRRT) peptide receptor radionuclide therapy; see also *Supplemental Table S 1* for tumor volume at ET start and PRRT start. ^1^ Sample codes A‒H can be found in the results section. Tumors from all treatment groups were snap-frozen and further processed for different molecular analyses including *Sstr2* promoter methylation, gene expression, and immunoblotting.

**Table 2:** SSTR2 binding constants in MPC and MTT cells treated with ET

Cell line	Treatment	K_d_ ± SEM (nmol/L)	*B*_max_ ± SEM (fmol/mg of protein)	*B*_max_ (binding sites/cell)	*B*_max_ (fold change)
MPC	Control	0.64 ± 0.12	157 ± 5.98	2.36 × 10^5^	-
MPC	VPA + DAC	0.77 ± 0.04	263 ± 3.45	3.96 × 10^5^	1.68‡
MTT	Control	1.24 ± 0.67	19.4 ± 3.11	2.92 × 10^4^	-
MTT	VPA + DAC	1.37 ± 0.35	63.4 ± 4.94	9.54 × 10^4^	3.27*

SSTR2 saturation binding of [^64^Cu]Cu-DOTA-TATE in cell homogenates was measured two days after final ET of cell cultures with VPA (10^-4^ mol/L) and DAC (10^-7^ mol/L) applied as two consecutive combination doses; *K*_d_ and *B*_max_ values were calculated from regression analysis (see *Supplemental Figure S 1B* for details); significance of differences (t-test): *P < 0.05; ‡ *P* < 0.01.

**Table 3:** Transcriptional effects and leading-edge genes of extracted KEGG pathways in MPC tumors responding differentially to [PRRT] and [ET + PRRT]

MPC tumors	[PRRT]↑↓ *vs*. [Control] (day 10)		[ET + PRRT]↑↓ *vs*. [Control] (day 10)		[ET]↑↓ *vs*. [Control] (day 4)	
	A*_V_*_ tumor 24h_ = 4.05 MBq/mL		A*_V_*_ tumor 24h_ = 4.48 MBq/mL			
**Extracted KEGG pathway**	nes (fdr)	Leading-edge genes (top 10)	nes (fdr)	Leading-edge genes (top 10)	nes (fdr)	Leading-edge genes
**Central carbon metabolism in cancer**	↓ ‒1.5 (0.10)	↓*Pdk1*, ↓*Slc2a1*	↑ 1.3 (0.23)	↑*Slc1a5*	↑ 1.8 (< 0.05)^ #^	↑*Hk2*
Arginine and proline metabolism	↓ ‒1.4 (0.21)	↓*Arg1*, ↓*P4ha1*	→	↑*P4ha3*	↑ 1.3 (0.22)	‒
Citrate cycle (TCA cycle)	↑ 1.8 (0.06)	‒	↑ 1.5 (0.09)	‒	→	‒
Glycolysis / Gluconeogenesis	→	↓*Pgk1*	↑ 1.3 (0.20)	‒	↑ 2.1 (< 0.05)^ #^	↑*Aldoc*, ↑*Hk2*
**Transcriptional misregulation in cancer**	→	↑*Ccnd2*, ↑*Tgfbr2*, ↑*Spint1*	↑ 1.6 (0.06)	↑*Ngfr*, ↑*Etv4*, ↑*Baiap3*, ↑*Runx1*, *↑*Tgfbr2*, *↑*Ccnd2*	↓ ‒1.3 (0.21)	↓*Igf1*
**Pathways in cancer**	↑ 1.6 (0.09)	↑*Cxcl12*, ↑*Mmp2*, ↑*Ccnd2*, ↑*Gli1*, ↑*Tgfbr2*	↑ 1.7 (< 0.05)	*↑*Mmp2*, *↑*Cxcl12*, ↑*Tcf7*, ↑*Hhip*, ↑*Jag2*, ↑*Axin2*, ↑*Runx1*, ↑*Lama5*, *↑*Tgfbr2*, *↑*Ccnd2, et seq.*	→	↓*Rac2*, ↓*Igf1*
Apoptosis	→	↑*Ptpn13*	↑ 1.3 (0.22)	↑*Ngfr*, *↑*Ptpn13*	↓ ‒1.6 (0.09)	↓*Ctsh*
Focal adhesion	↑ 1.6 (0.09)	↑*Tnxb*, ↑*Vwf*, ↑*Ccnd2*, ↑*Flnc*	↑ 1.8 (< 0.05)	↑*Tnc*, ↑*Flnc*, ↑*Col1a2*, ↑*Col1a1*, ↑*Col6a2*, ↑*Lama5*, *↑*Ccnd2*, ↑*Thbs2*, ↑*Col6a1*	↓ ‒1.3 (0.22)	↓*Spp1*, ↓*Rac2*, ↓*Igf1*
HIF-1 signaling pathway	↓ ‒1.8 (< 0.05)	↓*Pgk1*, ↓*Pdk1*, ↓*Egln3*, ↓*Slc2a1*	→	*↓*Egln3*	↑ 1.5 (0.10)	↑*Hk2*
mTOR signaling pathway	↓ ‒1.4 (0.15)	↓*Rragd*	→	‒	↑ 1.6 (0.07)	‒
Notch signaling pathway	↑ 1.5 (0.13)	‒	↑ 1.7 (< 0.05)	↑*Jag2*	→	‒
PPAR signaling pathway	↑ 2.0 (< 0.05)	↑*Plin4*, ↑*Fabp4*, ↑*Scd1*	↑ 1.5 (0.07)	*↑*Scd1*	→	↓*Lpl*, ↓*Pltp*, ↓*Cd36*
TGF-beta signaling pathway	→	↑*Dcn*, ↑*Tgfbr2*, ↑*Bambi*	↑ 1.6 (< 0.05)	↑*Nbl1*, ↑*Id1*, ↑*Dcn*, *↑*Tgfbr2*, ↑*Ltbp1*	→	‒
VEGF signaling pathway	→	‒	↑ 1.4 (0.13)	‒	↓ ‒1.4 (0.17)	↓*Rac2*, ↓*Mapkapk3*
Wnt signaling pathway	↑ 1.7 (0.07)	↑*Sfrp2*, ↑*Ccnd2*, ↑*Bambi*	↑ 2.0 (< 0.05)	↑*Tcf7*, ↑*Serpinf1*, ↑*Nfatc4*, ↑*Axin2*, *↑*Ccnd2*	→	↓*Rac2*
**DNA replication and repair**						
DNA replication	↑ 1.5 (0.10)	‒	→	‒	↓ ‒2.3 (< 0.05)	‒
Fanconi anemia pathway	→	‒	↓ ‒1.5 (0.11)	↓*Polk*	↓ ‒1.5 (0.13)^ #^	‒
Homologous recombination	→	‒	↓ ‒1.4 (0.14)	‒	→	‒

mRNA sequencing reported for sub-cohorts (*n* = 3); threshold for pathway enrichment: fdr < 0.25; thresholds for differentially expressed leading-edge genes: *P*_adj_ < 0.05, *log_2_* fold change ≥ |1|, SEM ≤ 0.5. (nes) normalized enrichment score; (fdr) false detection rate; (↑↓) up/down-regulation; (→) no change; * gene response shared with [PRRT]; ^#^ pathway response shared with [ET+PRRT].

**Table 4:** Transcriptional effects and leading-edge genes of extracted KEGG pathways in MTT tumors responding differentially to [PRRT] and [ET + PRRT]

MTT tumors	[PRRT]↑↓ *vs*. [Control] (day 10)		[ET + PRRT]↑↓ *vs*. [Control] (day 10)		[ET]↑↓ *vs*. [Control] (day 4)	
	A*_V_*_ tumor 24h_ = 0.44 MBq/mL		A*_V_*_ tumor 24h_ = 0.64 MBq/mL			
**Extracted KEGG pathway**	nes (fdr)	Leading-edge genes (top 10)	nes (fdr)	Leading-edge genes (top 10)	nes (fdr)	Leading-edge genes
**Central carbon metabolism in cancer**	↓ ‒1.4 (0.11)	↓*Hk2*, ↓*Pdk1*, ↓*Slc2a1*	↓ ‒1.5 (0.09)	*↓*Pdk1*, *↓*Slc2a1*, ↓*Pfkp*, ↓*Pfkl*, ↓*Pgam1*, ↓*Ldha*	↓ ‒2.0 (< 0.05)^ #^	↓*Hk2*
Citrate cycle (TCA cycle)	↑ 1.5 (0.13)	‒	↑ 1.7 (< 0.05)	‒	↑ 1.3 (0.16)^ #^	‒
Glycolysis/Gluconeogenesis	↓ ‒1.8 (< 0.05)	↓*Hk2*	↓ ‒1.8 (0.07)	↓*Aldoa*, ↓*Pfkp*, ↓*Gpi1*, ↓*Pfkl*, ↓*Aldoc*, ↓*Pgam1*, ↓*Ldha*, ↓*Pgk1*	↓ ‒1.7 (< 0.05)^ #^	↓*Hk2*, **↓*Aldoc*
Oxidative phosphorylation	↑ 1.4 (0.15)	‒	→	↑*Atp6v0d2*	↑ 2.5 (< 0.05)	↑*Atp6v0d2*, ↑*Cox6b2*
Pentose phosphate pathway	↓ ‒1.5 (0.06)	‒	↓ ‒1.6 (0.06)	↓*Aldoa*, ↓*Pfkp*, ↓*Gpi1*, ↓*Pfkl*, ↓*Aldoc*	↓ ‒1.5 (< 0.05)^ #^	↓*Aldoc*
**Pathways in cancer**	↑ 1.4 (0.12)	↑*Adcy8*, ↑*Jag2*, ↑*Tcf7*, ↑*Csf1r*, ↑*Mgst1*, ↑*Tgfbr2*, ↑*Igf1*, ↑*Cxcl12*, ↑*Adcy7*, ↑*Ccnd2, et seq.*	↑ 1.3 (0.16)	↑*Csf2rb2*, *↑*Mgst1*, ↑*Cebpa*, *↑*Csf1r*, *↑*Ccnd2*, ↑*Jag1*, ↑*Gnai1*, *↑*Tgfbr2*, ↑*Kitl*, *↑*Igf1, et seq.*	↓ ‒1.5 (< 0.05)	↓*Dcc*
Focal adhesion	↑ 1.3 (0.16)	↑*Tnxb*, ↑*Igf1*, ↑*Ccnd2*, ↑*Pdgfb*, ↑*Rac2*, ↑*Fyn*, ↑*Pdgfa*	↑ 1.3 (0.17)	*↑*Tnxb*, ↑*Vwf*, *↑*Ccnd2*, *↑*Igf1*, ↑*Lama3*, ↑*Itgb4*, ↑*Parvg*, *↑*Rac2*, ↑*Vav3, et seq.*	↓ ‒1.7 (< 0.05)	‒
HIF-1 signaling pathway	↓ ‒1.6 (0.05)	↓*Hk2*, ↓*Egln3*, ↓*Vegfa*, ↓*Pdk1*, ↓*Slc2a1*	↓ ‒1.8 (0.05)	*↓*Pdk1*, *↓*Vegfa*, *↓*Slc2a1*, ↓*Aldoa*, ↓*Pfkl*, ↓*Ldha*, ↓*Pgk1*	↓ ‒1.6 (< 0.05)^ #^	↓*Hk2*
Notch signaling pathway	↑ 1.4 (0.14)	↑*Jag2*	→	↑*Jag1*, ↑*Lfng*	↓ ‒1.6 (< 0.05)	‒
PPAR signaling pathway	→	↑*Pltp*	↑ 1.8 (< 0.05)	↑*Plin4*, ↑*Lpl*, *↑*Pltp*, ↑*Cd36*, ↑*Fabp4*	→	**↑*Lpl*, **↑*Pltp*, **↑*Cd36*, ↑*Nr1h3*
TGF-beta signaling pathway	↑ 1.4 (0.15)	↑*Neo1*, ↑*Dcn*, ↑*Tgfbr2*, ↑*Tgfb2*, ↑*Bmpr1b*, ↑*Smad3*	→	*↑*Bmpr1b*, *↑*Tgfbr2*, ↑*Bambi*	↓ ‒1.7 (< 0.05)	↓*Inhba*
**DNA replication and repair**						
Mismatch repair	↑ 1.4 (0.15)	‒	↑ 1.8 (< 0.05)	‒	↑ 2.1 (< 0.05)^ #^	‒

mRNA sequencing reported for treatment sub-cohorts (*n* = 3); threshold for pathway enrichment: fdr < 0.25; thresholds for differentially expressed leading-edge genes: *P*_adj_ < 0.05, *log_2_* fold change ≥ |1|, SEM ≤ 0.5. (nes) normalized enrichment score; (fdr) false detection rate; (↑↓) up/down-regulation; (→) no change; * gene response shared with [PRRT]; **gene response shared with [ET+PRRT]; ^#^ pathway response shared with [ET+PRRT].

## Abbreviations

A*_V_*: volume activity resp. activity concentration
CT: X-ray computed tomography
DAC: *5*-Aza-*2'*-deoxycytidine
DNMT: DNA-N-methyltransferase
ET: epigenetic treatment
GSEA: gene set enrichment analysis
HDAC: histone deacetylase
LD_50_: half-maximal lethal dose
MPC: mouse pheochromocytoma
MTT: mouse (MPC) tumor tissue-derived
PCC/PGL: pheochromocytoma and paraganglioma
PET: positron emission tomography
PRRT: peptide receptor radionuclide therapy
SPECT: single-photon emission computed tomography
SSTR2: somatostatin type 2 receptor
SUV: standardized uptake value
TATE: (Tyr^3^)octreotate
VPA: valproic acid
