# Supplementary material for: Epigenetic drugs in somatostatin type 2 receptor radionuclide theranostics and radiation transcriptomics in mouse pheochromocytoma models
Source: Theranostics. 2023 Jan 1;13(1):278–94. doi: 10.7150/thno.77918 (PMC9800739; doi:10.7150/thno.77918)
Supplement: Supplementary file 1 — Supplementary methods, figures and tables. [file thnov13p0278s1.pdf]

Epigenetic drugs in somatostatin type 2 receptor radionuclide theranostics and radiation transcriptomics in mouse pheochromocytoma models

– SUPPLEMENTAL INFORMATION –

Martin Ullrich[1], Susan Richter[2], Josephine Liers[1,2], Stephan Drukewitz[3,4], Markus Friedemann[2], Jörg Kotzerke[5], Christian G. Ziegler[6], Svenja Nölting[7,8], Klaus Kopka[1,9,10,11], and Jens Pietzsch[1,9]

[1] Helmholtz-Zentrum Dresden-Rossendorf, Institute of Radiopharmaceutical Cancer Research, Department of Radiopharmaceutical and Chemical Biology, Dresden, Germany
[2] University Hospital Carl Gustav Carus at the Technische Universität Dresden, Institute of Clinical Chemistry and Laboratory Medicine, Dresden, Germany
[3] National Center For Tumor Diseases/University Cancer Center Dresden, Core Unit for Molecular Tumor Diagnostics, Dresden, Germany
[4] University of Leipzig Medical Center, Institute of Human Genetics, Leipzig, Germany
[5] University Hospital Carl Gustav Carus at the Technische Universität Dresden, Klinik und Poliklinik für Nuklearmedizin, Dresden, Germany
[6] University Hospital Carl Gustav Carus at the Technische Universität Dresden, Department of Medicine III, Dresden, Germany
[7] University Hospital Zurich (USZ) and University of Zurich (UZH), Department of Endocrinology, Diabetology and Clinical Nutrition, Zurich, Switzerland
[8] University Hospital, LMU Munich, Department of Medicine IV, Munich, Germany
[9] Technische Universität Dresden, School of Science, Faculty of Chemistry and Food Chemistry, Dresden, Germany
[10] German Cancer Consortium (DKTK), Partner Site Dresden, Dresden, Germany
[11] National Center for Tumor Diseases (NCT), Partner Site Dresden, University Cancer Center (UCC), Dresden, Germany

Abbreviated Title: Epigenetic SSTR2 modulation in PCC/PGL

Corresponding author and person to whom reprint requests should be addressed:

Dr. Martin Ullrich

Helmholtz-Zentrum Dresden-Rossendorf

Institute of Radiopharmaceutical Cancer Research

Bautzner Landstraße 400, 01328 Dresden, Germany

Phone: +49-351-2604046, Fax: +49-351-26012622

E-mail: m.ullrich@hzdr.de

Abbreviations: A_V: volume activity resp. activity concentration; CT: X-ray computed tomography; DAC: *5*-Aza-*2′*-deoxycytidine; DNMT: DNA-N-methyltransferase; ET: epigenetic treatment; GSEA: gene set enrichment analysis; HDAC: histone deacetylase; LD_{50}: half-maximal lethal dose; MPC: mouse pheochromocytoma; MTT: mouse (MPC) tumor tissue-derived; PCC/PGL: pheochromocytoma and paraganglioma; PET: positron emission tomography; PRRT: peptide receptor radionuclide therapy; SPECT: single-photon emission computed tomography; SSTR2: somatostatin type 2 receptor; SUV: standardized uptake value; TATE: (Tyr^3)octreotate; VPA: valproic acid

Table of Content

1 Additional Methods.. 4

1.1 Preparation of epigenetic drugs...4

1.2 Radionuclide production and supply 4

1.3 Tumor volumes in animal cohorts 4

1.4 PET imaging and quantitative image analysis 5

1.5 SPECT imaging and quantitative image analysis 6

1.6 *Sstr2* promoter methylation analysis..6

1.7 Pre-selection of KEGG pathways for gene set enrichment analysis..........7

1.8 Real-time RT-PCR...7

2 Additional Results 9

2.1 [^{68}Ga]Ga-DOTA-TATE and [^{64}Cu]Cu-DOTA-TATE binding in response to epigenetic drugs in vitro..9

2.2 [^{64}Cu]Cu-DOTA-TATE uptake of allograft tumors in response to epigenetic drugs 10

2.3 [^{177}Lu]Lu-DOTA-TATE uptake and growth of allograft tumors in response to epigenetic drugs 17

2.4 Status of *Sstr2*/SSTR2 in allograft tumors in response to epigenetic drugs and [^{177}Lu]Lu-DOTA-TATE 20

2.5 Transcriptional responses of allograft tumors to epigenetic drugs and [^{177}Lu]Lu-DOTA-TATE – all genes and gene sets included...................23

2.6 Transcriptional responses of allograft tumors to epigenetic drugs and [^{177}Lu]Lu-DOTA-TATE – pre-selected gene sets involved in cancer and radiation resistance..26

1 Additional Methods

1.1 Preparation of epigenetic drugs

For *in vitro* application, VPA was freshly dissolved in cell culture medium and added to the cells at final concentrations between 10^{-5} and 10^{-2} mol/L. DAC was dissolved in H_2O to obtain a 10^{-4} mol/L stock solution that was stored at –20 °C. For each experiment, aliquots of the DAC stock were thawed and added to the cells at final concentrations between 10^{-8} and 10^{-5} mol/L.

For *in vivo* application, VPA was dissolved at 0.26 mol/L in Dulbecco's phosphate-buffered saline. DAC was dissolved at 0.04 mol/L in acetic acid (9.6 mol/L) to obtain a stock solution that was further diluted to 6.6×10^{-4} mol/L in Dulbecco's phosphate buffered. All solutions were adjusted to pH 7.2 using small amounts of aqueous NaOH (2.8 mol/L), sterile-filtered, and frozen at –20 °C.

1.2 Radionuclide production and supply

The radionuclide ^{68}Ga ($[^{68}Ga]GaCl_3$ dissolved in 1 mol/L HCl) was obtained from the commercial $^{68}Ge/^{68}Ga$-Generator IGG 100-50M (Eckert und Ziegler). The radionuclide ^{64}Cu ($[^{64}Cu]CuCl_2$ dissolved in 0.01 mol/L HCl) was produced at the Helmholtz-Zentrum Dresden-Rossendorf on the cyclotron TR-Flex (Advanced Cyclotron Systems Inc., Richmond, Canada) by a $^{64}Ni(p,n)^{64}Cu$ nuclear reaction. The radionuclide ^{177}Lu (EndolucinBeta®, non-carrier added $[^{177}Lu]LuCl_3$ dissolved in 0.04 mol/L HCl) was purchased from ITM (Isotope Technologies München AG, München, Germany).

1.3 Tumor volumes in animal cohorts

Animals with higher initial tumor volume were specifically included in cohorts that received epigenetic drugs in order to compensate for the growth-reducing effects of ET and to match tumor volumes as closely as possible across cohorts at the time of radiopharmaceutical injection

(Table S 1). The variation in tumor volumes at a specific time point can be explained by different tumor formation times, while tumor growth rates were largely similar. Differences in tumor volume between the treatment groups were comparable to the overall variation observed in the entire model cohort.

Table S 1: Tumor volumes of MPC and MTT allograft mice included in treatment groups; (ET_{start}) day 0 of the treatment schedule when animals received the first dose of epigenetic drugs; (ET_{start} + 4 d ≙ PET_{start} / $PRRT_{start}$) day 4 of the investigation when animals received a single dose of radiopharmaceutical to perform PET imaging or PRRT depending on the radiopharmaceutical applied; data presented as means ± SEM

Cohort label	ET	Radiopharmaceutical	V_{tumor} (cm^3) ET_{start}	V_{tumor} (cm^3) ET_{start} + 4 d ≙ PET_{start} / $PRRT_{start}$
		MPC allograft model − PET and biodistribution		
[Control]	Vehicle (PBS)	[^{64}Cu]Cu-DOTA-TATE	0.18 ± 0.12	0.28 ± 0.13
[ET_{VPA}]	VPA	[^{64}Cu]Cu-DOTA-TATE	0.27 ± 0.13	0.46 ± 0.23
[ET_{DAC}]	DAC	[^{64}Cu]Cu-DOTA-TATE	0.39 ± 0.16	0.57 ± 0.18
[$ET_{VPA + DAC}$]	VPA + DAC	[^{64}Cu]Cu-DOTA-TATE	0.51 ± 0.17	0.81 ± 0.20
		MTT allograft model − PET and biodistribution		
[Control]	Vehicle (PBS)	[^{64}Cu]Cu-DOTA-TATE	0.13 ± 0.02	0.42 ± 0.08
[ET_{VPA}]	VPA	[^{64}Cu]Cu-DOTA-TATE	0.14 ± 0.05	0.35 ± 0.05
[ET_{DAC}]	DAC	[^{64}Cu]Cu-DOTA-TATE	0.23 ± 0.07	0.24 ± 0.06
[$ET_{VPA + DAC}$]	VPA + DAC	[^{64}Cu]Cu-DOTA-TATE	0.28 ± 0.08	0.37 ± 0.11
		MPC allograft model − PRRT, SPECT, and gene expression		
[Control]	Vehicle (PBS)	w/o	0.17 ± 0.03	0.39 ± 0.03
[ET]	VPA + DAC	w/o	0.25 ± 0.05	0.44 ± 0.10
[PRRT]	Vehicle (PBS)	[^{177}Lu]Lu-DOTA-TATE	0.09 ± 0.01	0.24 ± 0.06
[ET + PRRT]	VPA + DAC	[^{177}Lu]Lu-DOTA-TATE	0.25 ± 0.02	0.48 ± 0.05
		MTT allograft model − PRRT, SPECT, and gene expression		
[Control]	Vehicle (PBS)	w/o	0.13 ± 0.02	0.54 ± 0.05
[ET]	VPA + DAC	w/o	0.37 ± 0.08	0.53 ± 0.06
[PRRT]	Vehicle (PBS)	[^{177}Lu]Lu-DOTA-TATE	0.12 ± 0.01	0.36 ± 0.04
[ET + PRRT]	VPA + DAC	[^{177}Lu]Lu-DOTA-TATE	0.34 ± 0.08	0.59 ± 0.08

1.4 PET imaging and quantitative image analysis

Small animal positron emission tomography (PET) was performed using the nanoPET/CT scanner (Mediso Medical Imaging Systems, Budapest, Hungary). Images were reconstructed using the Tera-Tomo™ three-dimensional (3D) algorithm using a voxel size of 0.4 mm and applying corrections for scatter, attenuation, and decay. Images were post-processed and analyzed using ROVER (ABX, Radeberg, Germany). Three-dimensional volumes of interest

(VOIs) were created (40–60 min frames) applying fixed thresholds for delineation of tumor (30%), muscle (0%), kidneys (25%), and liver (35%).

1.5 SPECT imaging and quantitative image analysis

Small animal single-photon emission computed tomography nanoSPECT/CT was performed using the nanoSPECT/CT scanner (Mediso Medical Imaging Systems) equipped with the APT62 aperture consisting of four M3 multi-pinhole collimators providing a 30×30 mm axial field of view (FOV). Photon emission was recorded using a frame time of 120 s (total scan time of 90 min) and binned within the 20% energy windows of the 56, 113, and 208 keV photopeaks. Images were reconstructed using the Tera-Tomo™ three-dimensional (3D) algorithm at high dynamic range using a voxel size of 0.4 mm and applying corrections for scatter and attenuation. Images were post-processed and analyzed using ROVER (ABX). Three-dimensional VOIs were created by applying a fixed threshold for delineation of tumor (20%), kidneys (15%), and liver (25%).

1.6 Sstr2 promoter methylation analysis

DNA from cell cultures and allografts was extracted using the DNeasy Blood and Tissue Kit (Qiagen, Venlo, The Netherlands) and treated with bisulfite using the EpiTect Fast DNA Bisulfite Kit (Qiagen, Hilden, Germany). Bisulfite-converted DNA was amplified using a primer pair covering 20 CpGs of the *Sstr2* promoter. Amplicon size (195bp) was confirmed on an agarose gel and the PCR product was sent for Sanger sequencing (Microsynth, Balbach, Switzerland). Electropherograms were compared to PCR products amplified from bisulfite-converted mouse Universal Methylated DNA Standard (Zymoresearch, Irvine CA, USA) and an unmethylated 900bp-DNA fragment. The latter was generated by amplification of genomic mouse DNA using primers annealing around the CpG island of *Sstr2*. Nucleotide sequences of primers are provided in (Table S 2).

Table S 2: Primer pairs used for *Sstr2* promoter methylation analysis

Target	Nucleotide sequence
Amplification of bisulfite-converted DNA	5’-AtTtTGtTtAtCGGGTttAAtAGGAtt-3’
	5’-CCTaTAaATCATTaACGCCCAaCC-3’
Generation of an unmethylated DNA fragment	5’-GGTTGGGCTGGGGCTGGGTC-3’
	5’- CCTCGAGCACTCGCTTCCCTGTG-3’

1.7 Pre-selection of KEGG pathways for gene set enrichment analysis

For investigations on transcriptional responses associated with ET and PRRT, 39 pathways were pre-selected from the KEGG database for gene set enrichment analysis. The latter included the following two categories: (1) pathways involved in cancer (‘pathways in cancer’ [mmu05200 and pathways therein]; ‘transcriptional misregulation in cancer’ [mmu05202]) and (2) pathways involved in the sensitivity to ionizing radiation (central carbon metabolism in cancer [mmu5230 and pathways therein]; DNA damage repair [mmu03030, mmu03410, mmu03420, mmu03430, mmu03440, mmu03450, mmu03460]; reactive oxygen species (ROS) defense [mmu00480]).

A specific subset of these enrichment pathways was extracted representing the additional effects of ET on the regular response to PRRT. These gene sets met two conditions: (i) enrichment in [ET + PRRT] *vs.* [PRRT], *and* at the same time (ii) overlapping with enrichment in [PRRT] *vs.* [Control] *or* with enrichment in [ET + PRRT] *vs.* [Control]. Differentially expressed leading-edge genes from the extracted enrichment pathways were reported.

1.8 Real-time RT-PCR

cDNA was prepared from mouse RNA using qScript cDNA Synthesis Kit (Quantabio, Beverly MA, USA) following the manufacturer’s recommendations. cDNA was diluted 1:2 and amplified with the PerfeCTa SYBR Green Super Mix Low Rox (Quantabio, Beverly MA, USA) on a CFX Connect Real-Time PCR Detection System (Bio-Rad, Hercules CA, USA) using primer pairs specific for *Sstr2, Chga, Actb*, and *Rpl19* (Table S 3). Amplicons were generated

in 40 cycles (95°C 5 sec, 60°C 10 sec) with 5 minutes at 95°C for initial denaturation and characterized by melting curve analysis and on an agarose gel.

Table S 3: Primer pairs used for real-time RT-PCR

Target gene	Nucleotide sequence
Sstr2	5’-CGCATGGTGTCCATCGTAGT-3’
	5’-GGATTGTGAATTGTCTGCCTTGA-3’
Chga	5’-CCAAGGTGATGAAGTGCGTC-3’
	5’-GGTGTCGCAGGATAGAGAGGA-3’
Actb	5’-GGCTGTATTCCCCTCCATCG-3’
	5’-CCAGTTGGTAACAATGCCATGT-3’
Rpl19	5’-ATATGGGCATAGGGAAGAGG-3’
	5’-CTGTCTGCCTTCAGCTTGT-3’

2 Additional Results

2.1 [^{68}Ga]Ga-DOTA-TATE and [^{64}Cu]Cu-DOTA-TATE binding in response to epigenetic drugs in vitro

In MPC cells, the radioligand assay showed impairment of [^{68}Ga]Ga-DOTA-TATE uptake at 37° C due to cytotoxic effects of VPA and DAC at concentrations of 10^{-3} mol/L and 5×10^{-7} mol/L, respectively. Hence the following experiments were performed with lower concentrations of the epigenetic drugs (Figure S 1A).

Binding assays with cell homogenates showed that ET, a combination of 10^{-4} mol/L VPA and 10^{-7} mol/L DAC, significantly increased the specific binding capacity for [^{64}Cu]Cu-DOTA-TATE in both MPC and MTT cells (Figure S 1B).

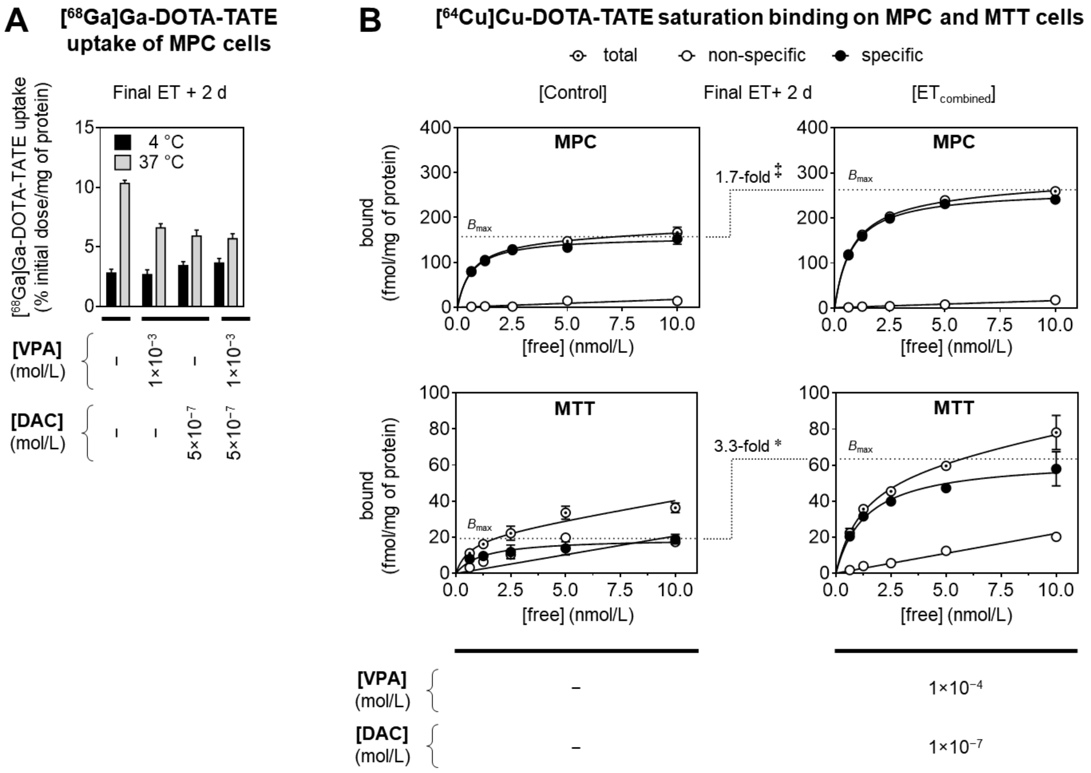

Figure S 1: SSTR2 radiotracer assays and effects of epigenetic drugs; (A) Decreased [^{68}Ga]Ga-DOTA-TATE uptake in MPC cells at 37° C after treatment with VPA and DAC at concentrations of 10^{-3} mol/L and 5×10^{-7} mol/L, respectively; **(B)** Saturation binding of [^{64}Cu]Cu-DOTA-TATE in homogenates of MPC and MTT cells treated with VPA and DAC at concentrations of 10^{-4} mol/L and 10^{-7} mol/L, respectively; (dotted lines) B_{max} values of SSTR2 binding sites; significance of differences (t-test): *$P < 0.05$; ‡ $P < 0.01$

2.2 [^{64}Cu]Cu-DOTA-TATE uptake of allograft tumors in response to epigenetic drugs

PET images of MPC and MTT allograft mice provide an overview over ET effects on the distribution of [^{64}Cu]Cu-DOTA-TATE in individual animals (Figure S 2). Quantitative image analysis showed the reduction of [^{64}Cu]Cu-DOTA-TATE uptake in MPC tumors and the stimulation of [^{64}Cu]Cu-DOTA-TATE in MTT tumors in response to ET. Extracted standardized uptake values (SUVmean, SUVmax) showed similar trends to reference tissue ratios (tumor/muscle). (Table S 4). Uptake values in tumors measured *ex vivo* (SUV and % initial dose/g tissue) confirmed these observations (Figure S 3).

Uptake of [^{64}Cu]Cu-DOTA-TATE in tumors was correlated with other parameters such as initial tumor, ET-induced reduction in tumor growth, and ET-induced changes in the biodistribution of the radiotracer. Tumor growth was reduced upon ET in both MPC and MTT allograft mice (Figure S 4A). Since correlation analyses did not show any relationship between tumor volume and the SUV (Figure S 4B), animals with tumor volumes of 0.05−1.4 cm^3 for MPC and 0.04−0.82 cm^3 for MTT were included in quantitative image analyses focusing on [^{64}Cu]Cu-DOTA-TATE uptake. A positive linear relationship between growth-reducing effects of ET and reduced SUVs in MPC tumors indicate that cytostatic effects of the epigenetic drugs contributed to the reduction of [^{64}Cu]Cu-DOTA-TATE uptake (Figure S 4C)**.**

Using the treatment protocol, epigenetic drugs showed no statistically relevant effects on [^{64}Cu]Cu-DOTA-TATE retention in blood as determined from areas under time-activity curves ($AUC_{0–60\ min}$) in the heart, nor did individual differences in activity retention in blood correlate with SUV changes in tumors (Figure S 5A–B).

Activity retention in the liver was significantly higher in DAC-treated animals; however, this effect showed no relationship with SUV changes in tumors (Figure S 5C–D). Activity in the

liver may also have resulted from [^{64}Cu]Cu^{2+} trans-chelation reactions and small amounts of free [^{64}Cu]Cu^{2+} ions (< 3%) remaining in the radiotracer preparation.

Activity in the renal cortex showed no statistically relevant differences between treatment groups nor did individual differences correlate with SUV changes in tumors (Figure S 5E–F). Some animals showed higher activity in the renal pelvis, resulting from activity in primary urine that has not yet been drained completely.

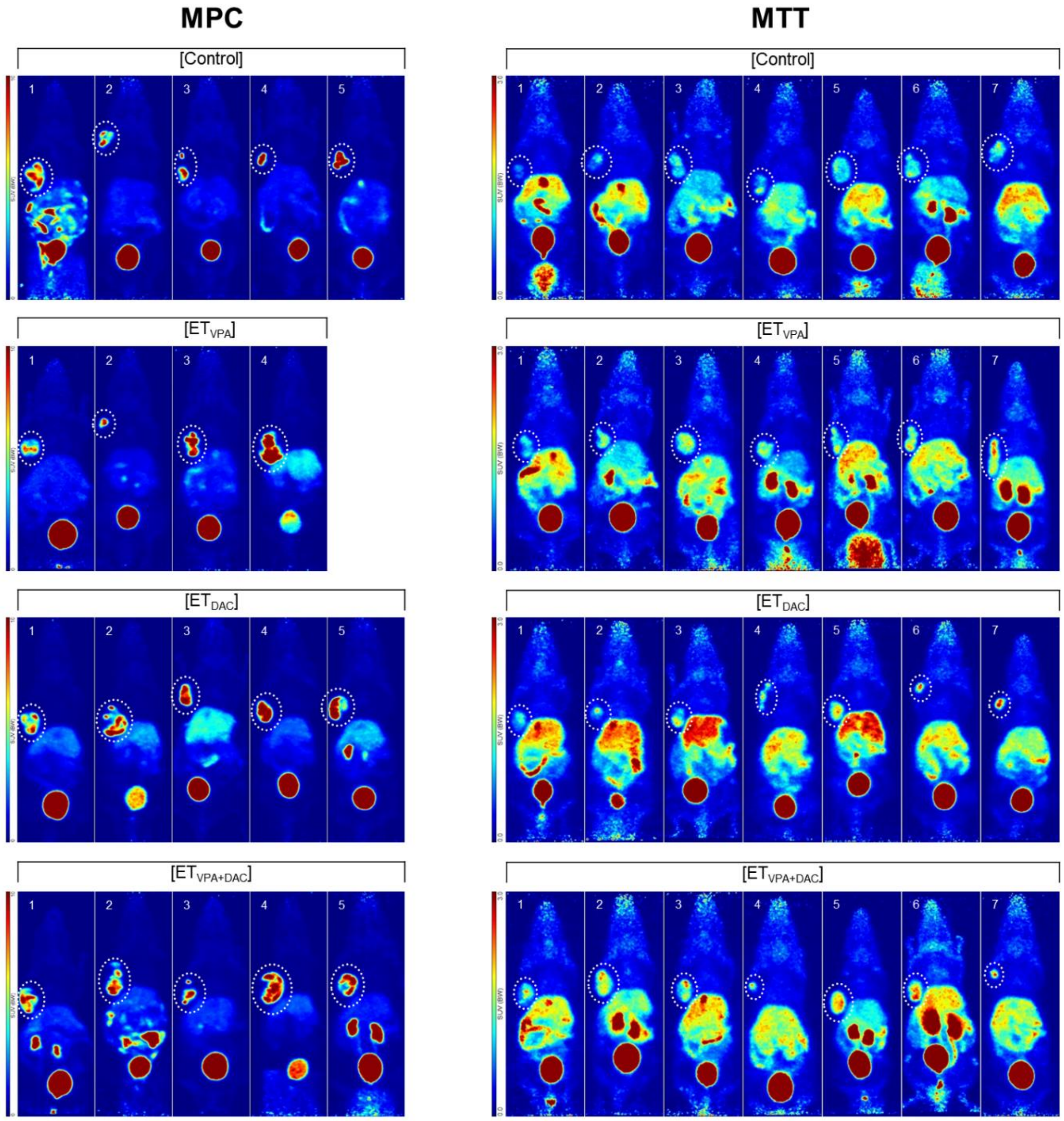

Figure S 2: PET images of [^{64}Cu]Cu-DOTA-TATE distribution in MPC and MTT allograft mice in response to epigenetic treatment; Maximum intensity projections presented with different SUV color scaling: MPC (0−10); MTT (0−3); ET: treatment with VPA (250 mg/kg) and DAC (1 mg/kg) as single and combination doses, respectively, on days −3 and 0; PET imaging with [^{64}Cu]Cu-DOTA-TATE (10 MBq/animal, equivalent to 0.25 nmol) on day 1 after final ET; MPC allograft mice [Control #1 and #5] and [ET VPA+DAC #2] showed activity hotspots within the intestinal loops resulting from accidental intake of contaminated bedding materials assimilated prior to PET scanning (between radiotracer injection and induction of anesthesia); (dotted regions) radiotracer uptake in tumors 40−60 min after injection; see Table S 4 for uptake values in tumors

Table S 4: Uptake of [^{64}Cu]Cu-DOTA-TATE in tumors of MPC and MTT allograft mice treated with epigenetic drugs; ET: treatment with VPA (250 mg/kg) and DAC (1 mg/kg) as single and combination doses, on days −3 and 0; PET imaging with [^{64}Cu]Cu-DOTA-TATE (10 MBq/animal, equivalent to 0.25 nmol) on day 1 after final ET; (SUV) standardized uptake values 40–60 min after injection of the radiotracer

Cohort label	Animals	SUVmean tumor	SUVmean ratio tumor / muscle	SUVmax tumor	SUVmax ratio tumor / muscle
			MPC allograft mice		
[Control]	1	5.71	61.5	12.5	62.4
	2	6.16	56.4	13.8	51.5
	3	8.04	85.8	19.2	83.4
	4	9.46	112	18.8	63.8
	5	10.3	103	22.7	106
	mean ± SEM	7.93 ± 0.89	83.8 ± 11.0	17.4 ± 1.89	73.3 ± 9.54
[ET $_{VPA}$]	1	4.22	60.0	8.80	33.0
	2	5.61	67.2	10.8	60.8
	3	8.55	91.2	19.7	78.3
	4	9.24	100	18.5	65.2
	mean ± SEM	6.91 ± 1.19	79.6 ± 9.53	14.5 ± 2.73	59.3 ± 9.54
[ET $_{DAC}$]	1	5.09	68.2	11.5	57.8
	2	6.74	96.7	13.9	65.6
	3	7.52	93.2	15.3	58.7
	4	7.99	80.6	15.2	69.1
	5	9.57	104	20.6	67.9
	mean ± SEM	7.38 ± 0.74	88.6 ± 6.39	15.3 ± 1.49	63.8 ± 2.35
[ET $_{VPA+DAC}$]	1	5.89	73.7	13.2	47.4
	2	6.11	82.3	13.2	66.9
	3	6.63	70.9	13.4	57.6
	4	7.04	79.7	15.2	63.3
	5	7.48	90.0	16.0	72.2
	mean ± SEM	6.63 ± 0.29	79.3 ± 3.36	14.2 ± 0.58	61.5 ± 4.24
			MTT allograft mice		
[Control]	1	0.12	1.32	0.25	1.09
	2	0.43	4.91	1.02	4.05
	3	0.54	8.63	1.33	4.52
	4	0.55	6.83	1.39	6.57
	5	0.60	7.64	1.53	9.62
	6	0.68	9.13	1.67	6.34
	7	0.68	8.43	1.63	8.38
	mean ± SEM	0.51 ± 0.07	6.70 ± 1.04	1.26 ± 0.19	5.80 ± 1.08
[ET $_{VPA}$]	1	0.34	3.20	0.69	3.3
	2	0.70	10.52	1.41	4.72
	3	0.71	7.63	1.61	8.25
	4	0.73	11.4	1.60	10.8
	5	0.74	7.80	1.62	5.31
	6 ^{R}	0.86	9.73	1.73	5.70
	7 ^{R}	1.06	9.95	2.23	6.06
	mean ± SEM	0.73 ± 0.08	8.60 ± 1.04	1.55 ± 0.17	6.30 ± 0.94
[ET $_{DAC}$]	1	0.38	4.91	0.83	4.01
	2	0.65	7.82	1.52	6.62
	3	0.77	8.27	1.66	5.31
	4 ^{R}	0.80	12.6	2.00	11.0
	5 ^{R}	0.93	11.5	1.88	7.68
	6 ^{R}	1.24	17.9	2.71	14.7
	7 ^{R}	1.37	21.3	2.72	18.9
	mean ± SEM	0.88 ± 0.13 *	12.1 ± 2.2 *	1.90 ± 0.25	9.74 ± 2.05
[ET $_{VPA+DAC}$]	1	0.74	8.72	1.68	7.80
	2 ^{R}	0.97	10.8	2.31	8.84
	3 ^{R}	0.99	9.47	2.44	10.7
	4 ^{R}	1.02	10.8	2.34	8.98
	5 ^{R}	1.19	13.1	2.34	11.8
	6 ^{R}	1.24	10.7	2.81	7.75
	7 ^{R}	1.27	16.6	2.88	15.0
	mean ± SEM	1.06 ± 0.07 #	11.5 ± 1	2.40 ± 0.15 ‡	10.1 ± 0.99

^{R} SUV responders to epigenetic treatment; responder thresholds were calculated from the SUVmean values of the [Control] cohorts + two times typical error (2×TE)

* Significance of differences compared to [Control]: *P < 0.05; ‡ P < 0.01; # P < 0.001

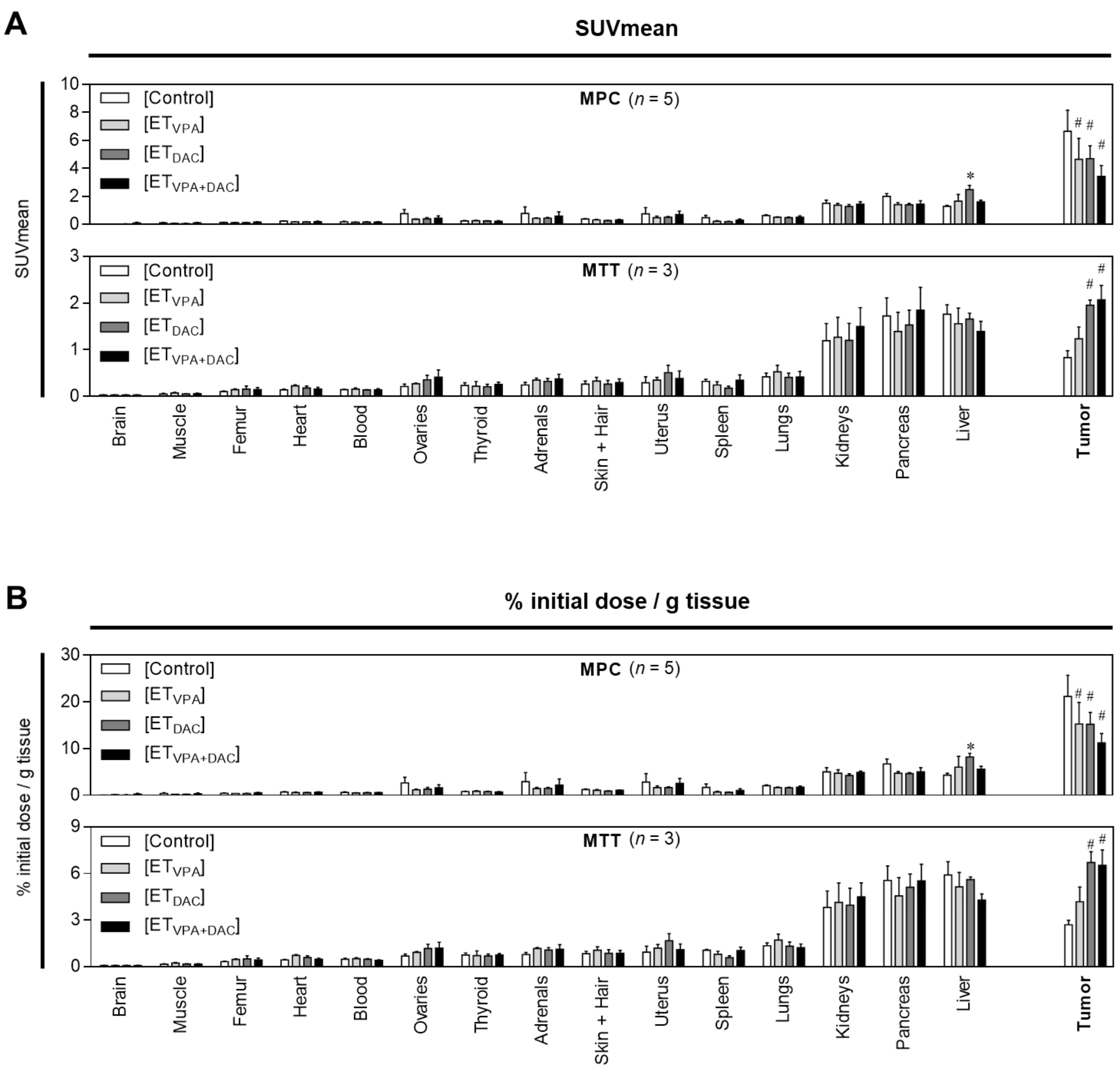

Figure S 3: Distribution of $[^{64}Cu]$Cu-DOTA-TATE in MPC and MTT allograft mice treated with epigenetic drugs as measured *ex vivo* in tissue samples; (A) Radiotracer distribution reported as standardized uptake values; **(B)** Radiotracer distribution reported as % initial dose/g tissue; both evaluation methods showed similar effects of ET with increased tumor uptake in MTT allograft mice only; significance of differences: $^{\#}$ $P < 0.001$

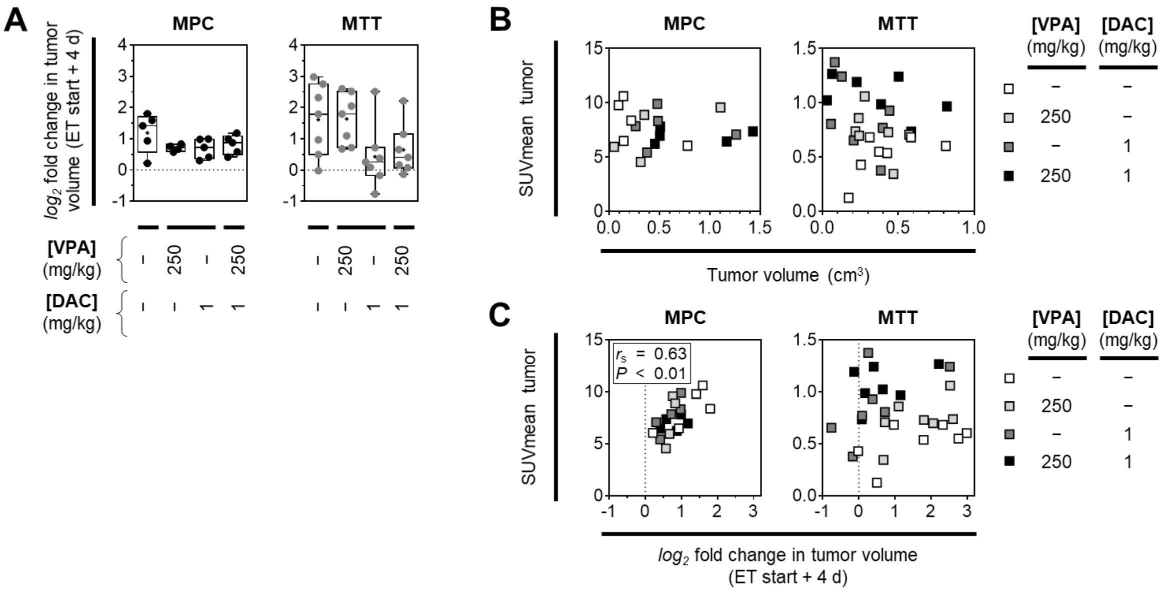

Figure S 4: Correlation analyses between uptake of [^{64}Cu]Cu-DOTA-TATE in tumors and tumor growth in MPC and MTT allograft mice treated with epigenetic drugs; (A) Changes in tumor volume in response to ET showing reduced growth in both MPC and MTT allograft mice; (*log₂* fold changes) number of volume doublings compared to ET start; **(B)** Correlation analyses showing independence of radiotracer uptake in tumors (SUVmean) from tumor volumes (cm^3) across all treatment groups; **(C)** Correlation analyses showing a positive linear relationship between the growth-reducing effects of ET and reduced radiotracer uptake (SUVmean) in MPC tumors

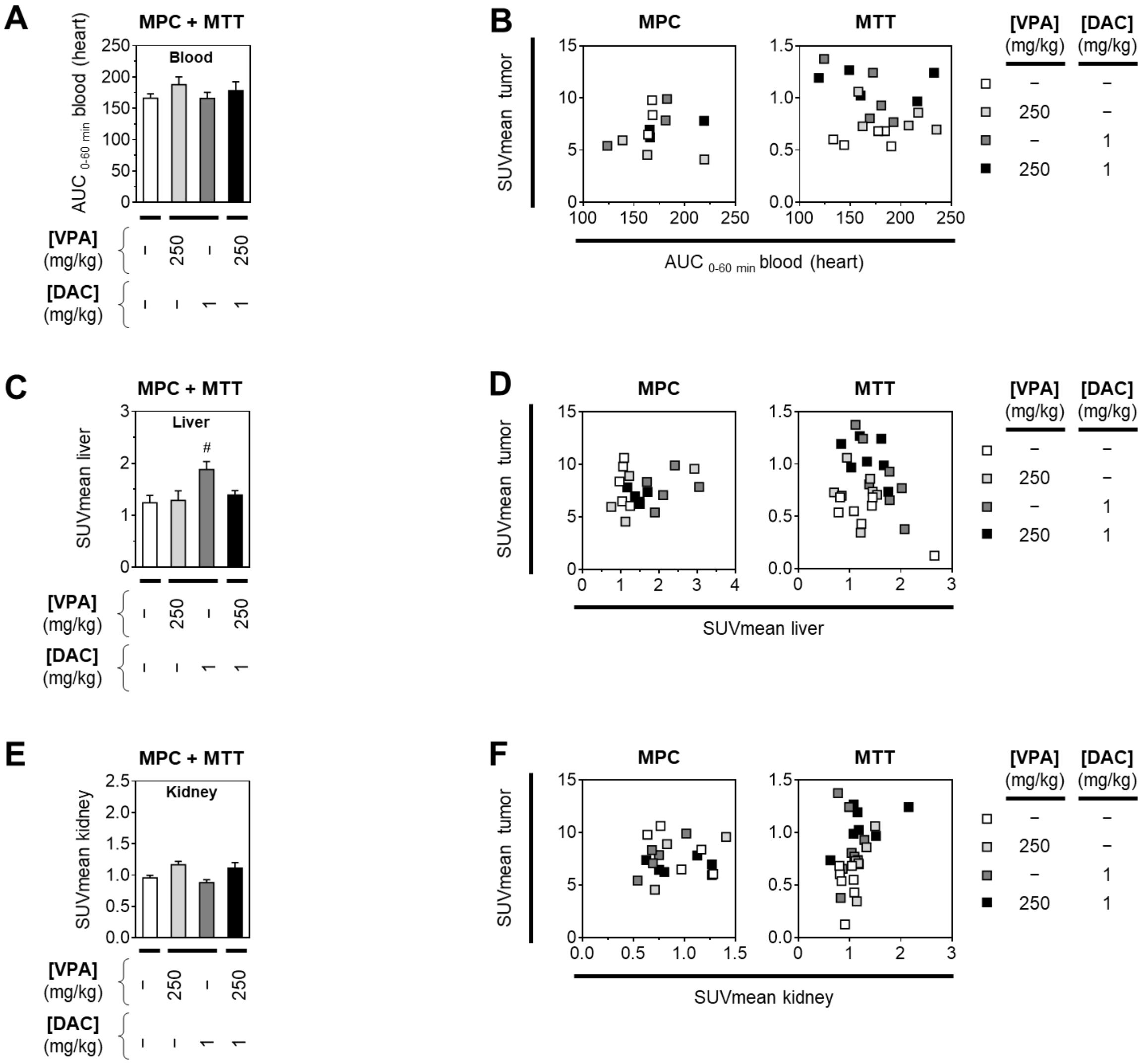

Figure S 5: Correlation analyses between uptake of [^{64}Cu]Cu-DOTA-TATE in tumors and retention in blood, liver, and kidneys in MPC and MTT allograft mice treated with epigenetic drugs; (A–B) Activity retention in blood determined from areas under time-activity curves ($AUC_{0\text{-}60\ min}$) in the heart and correlation with tumor uptake; **(C–D)** Activity retention in the liver and correlation with tumor uptake; **(E–F)** Activity retention in the renal cortex and correlation with tumor uptake; significance of differences: $^{\#}$ $P < 0.001$

2.3 [^{177}Lu]Lu-DOTA-TATE uptake and growth of allograft tumors in response to epigenetic drugs

SPECT images of MPC and MTT allograft mice provide an overview over ET effects on the distribution of [^{177}Lu]Lu-DOTA-TATE in individual animals (Figure S 6). Excretion of the radiotracer via the renal pathway was associated with some retention of activity in the renal cortex. Small amounts of free [^{177}Lu]Lu^{3+} ions (< 5%) that remained in the radiotracer preparation contributed to retention of activity in liver and bones, in particular in joints. Quantitative image analysis showed no effect of ET on [^{177}Lu]Lu-DOTA-TATE uptake in MPC tumors but a higher uptake in MTT tumors. Analyses of VOI-averaged activity concentrations (A_V mean) and activity hotspots (A_V max) in tumors showed similar trends (Table S 5).

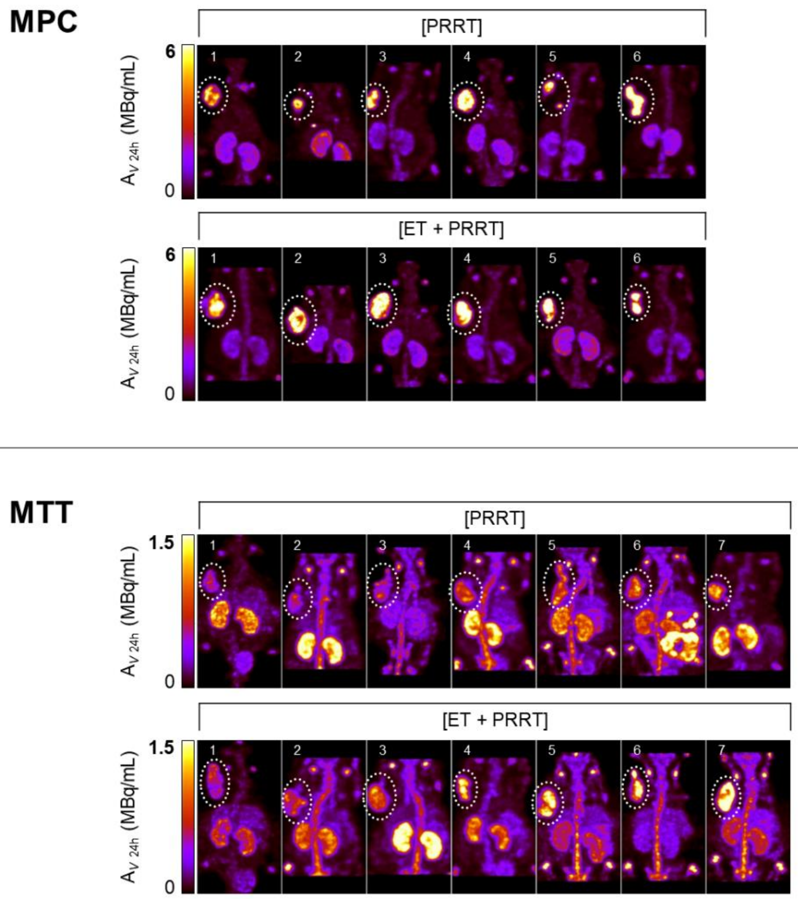

Figure S 6: SPECT images of [^{177}Lu]Lu-DOTA-TATE distribution in MPC and MTT allograft mice and effects of epigenetic treatment; Maximum intensity projections presented at different A_V color scaling: MPC (0−6 MBq/mL); MTT (0−1.5 MBq/mL); ET: treatment with VPA (250 mg/kg) and DAC (1 mg/kg) as combination doses on days −4 and −1; PRRT: treatment with [^{177}Lu]Lu-DOTA-TATE (70 MBq/animal, equivalent to 1.2 nmol) as a single dose on day 0; quantitative SPECT imaging on day 1; ($A_{V\,24\,h}$) activity concentration of radionuclide drug 24 hours after injection; (dotted regions); MTT allograft mouse [PRRT #6] showed activity hotspots within the stomach and the intestinal loops resulting from accidental intake of contaminated bedding materials assimilated prior to image recording (between injection of radiopharmaceutical and initiation of anesthesia); see Table S 5 for tumor uptake values and follow-up of tumor growth in individual animals

Table S 5: Uptake of [^{177}Lu]Lu-DOTA-TATE in tumors and follow-up of tumor growth in MPC and MTT allograft mice treated with epigenetic drugs; ET: treatment with VPA (250 mg/kg) and DAC (1 mg/kg) as combination doses on days −4 and −1; PRRT: treatment with [^{177}Lu]Lu-DOTA-TATE (70 MBq/animal, equivalent to 1.2 nmol) as a single dose on day 0; ($A_{V\,24\,h}$) activity concentration of the radionuclide drug 24 hours after injection; mRNA of tumors was obtained from the sub-cohorts A−H

Cohort label	Animals entire cohort	Animals sub-cohort (mRNA samples)[1]	A_V mean $_{24h}$ tumor (MBq/mL)	A_V max $_{24h}$ tumor (MBq/mL)	Follow-up tumor growth (days after ET start)
[Control]	1	A1	−	−	4
	2	A2	−	−	4
	3	A3	−	−	4
[ET]	1	B1	−	−	4
	2	B2	−	−	4
	3	B3	−	−	4
[PRRT]	1	−	3.10	7.13	10
	2	−	3.31	8.31	10
	3	C3	3.68	8.36	10
	4	C4	4.14	10.1	10
	5	C5	4.33	10.1	10
	6	−	5.56	13.2	10
	mean ± SEM		4.02 ± 0.36	9.53 ± 0.87	
		mean ± SEM	4.05 ± 0.19	9.52 ± 0.58	
[ET + PRRT]	1	−	3.32	8.11	10
	2	−	3.76	10.0	10
	3	D3	4.13	10.5	10
	4	D4	4.38	11.6	10
	5	D5	4.93	13.4	10
	6 [R]	−	7.22	18.0	11[E]
	mean ± SEM		4.62 ± 0.57	11.9 ± 1.41	
		mean ± SEM	4.48 ± 0.24	11.8 ± 0.85	
[Control]	1	E2	−	−	4
	2	E2	−	−	4
	3	E3	−	−	4
[ET]	1	F1	−	−	4
	2	F2	−	−	4
	3	F3	−	−	4
[PRRT]	1	−	0.33	0.92	7[A]
	2	G2	0.36	1.03	10
	3	G3	0.39	1.17	10
	4	G4	0.56	1.36	10
	5	−	0.59	1.53	10
	6	−	0.60	1.62	7[A]
	7	−	0.70	1.66	10
	mean ± SEM		0.50 ± 0.05	1.33 ± 0.19	
	−	mean ± SEM	0.44 ± 0.06	1.19 ± 0.10	
[ET + PRRT]	1	−	0.39	1.04	10
	2	H2	0.47	1.32	10
	3	H3	0.56	1.45	10
	4 [R]	H4	0.88	2.22	10
	5 [R]	−	0.93	2.71	18[E]
	6 [R]	−	1.04	3.17	18[E]
	7 [R]	−	1.23	3.34	18[E]
	mean ± SEM		0.79 ± 0.12 *	2.18 ± 0.35	
		mean ± SEM	0.64 ± 0.12	1.66 ± 0.28	

[1] Sample codes A–H can be found in the results section.

[R] A_V responders to epigenetic treatment; responder thresholds were calculated from the mean A_V values of the [PRRT] cohorts + two times typical error (2×TE); significance of differences compared to [PRRT]: *P < 0.05

[A] Aborted follow-up due to incidental death during imaging procedure

[E] Extended follow-up in selected animals presenting with highest initial activity concentrations in tumor

2.4 Status of Sstr2/SSTR2 in allograft tumors in response to epigenetic drugs and [^{177}Lu]Lu-DOTA-TATE

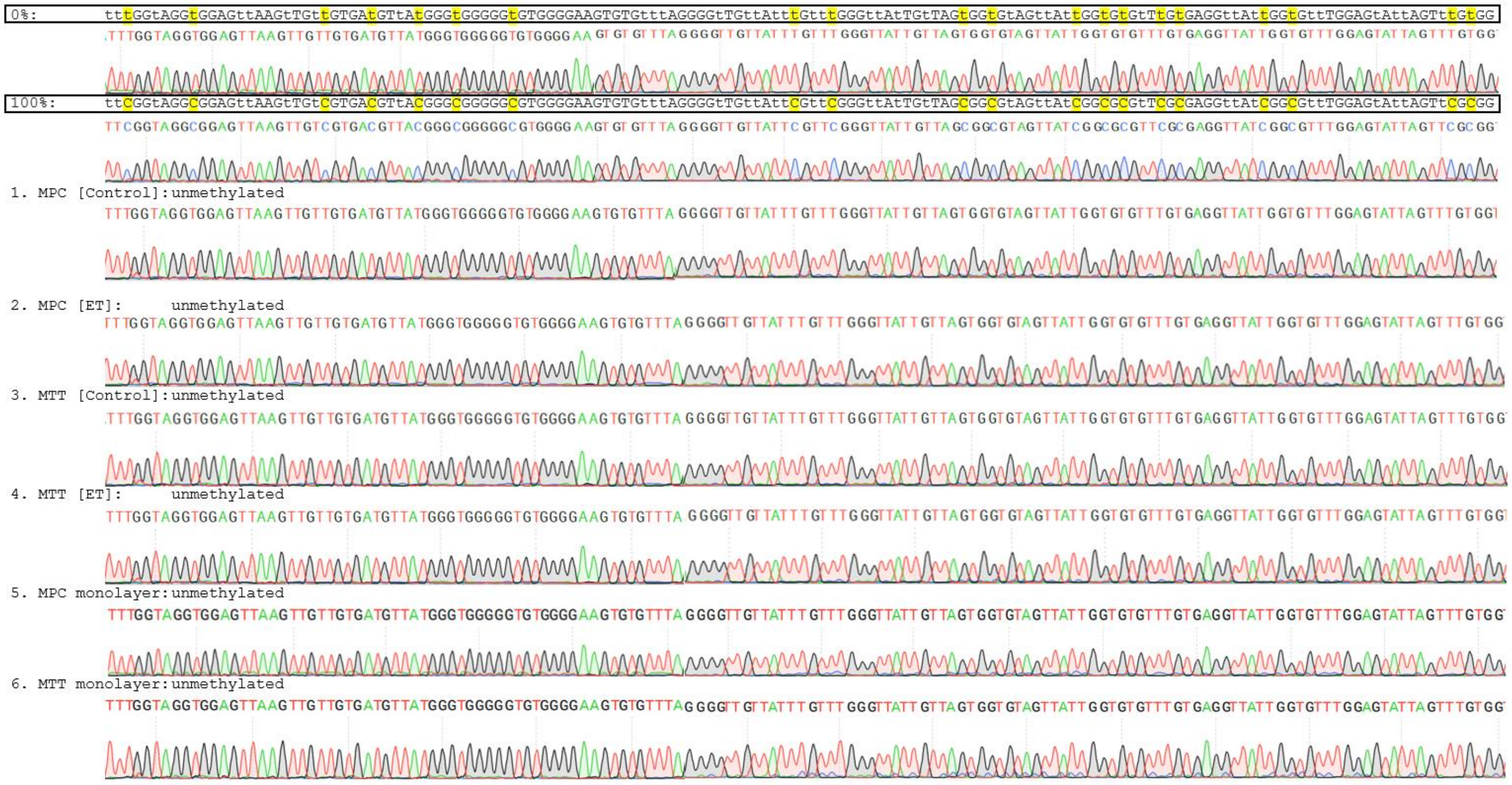

Figure S 7: *Sstr2* promoter methylation; ET: treatment with VPA (250 mg/kg) and DAC (1 mg/kg) as combination doses on days 0 and 3; Genomic DNA from allograft tumors (1-4) or monolayer cultures (5-6) was extracted and treated with bisulfite. PCR products spanning 20 CpGs (highlighted in yellow) of the *Sstr2* promoter (amplified region 11:113510045-113510239,GRCm39) were generated and Sanger sequenced (forward and reverse) together with methylated (100%) and unmethylated (0%) control DNA. Representative examples of three different allografts per group are depicted.

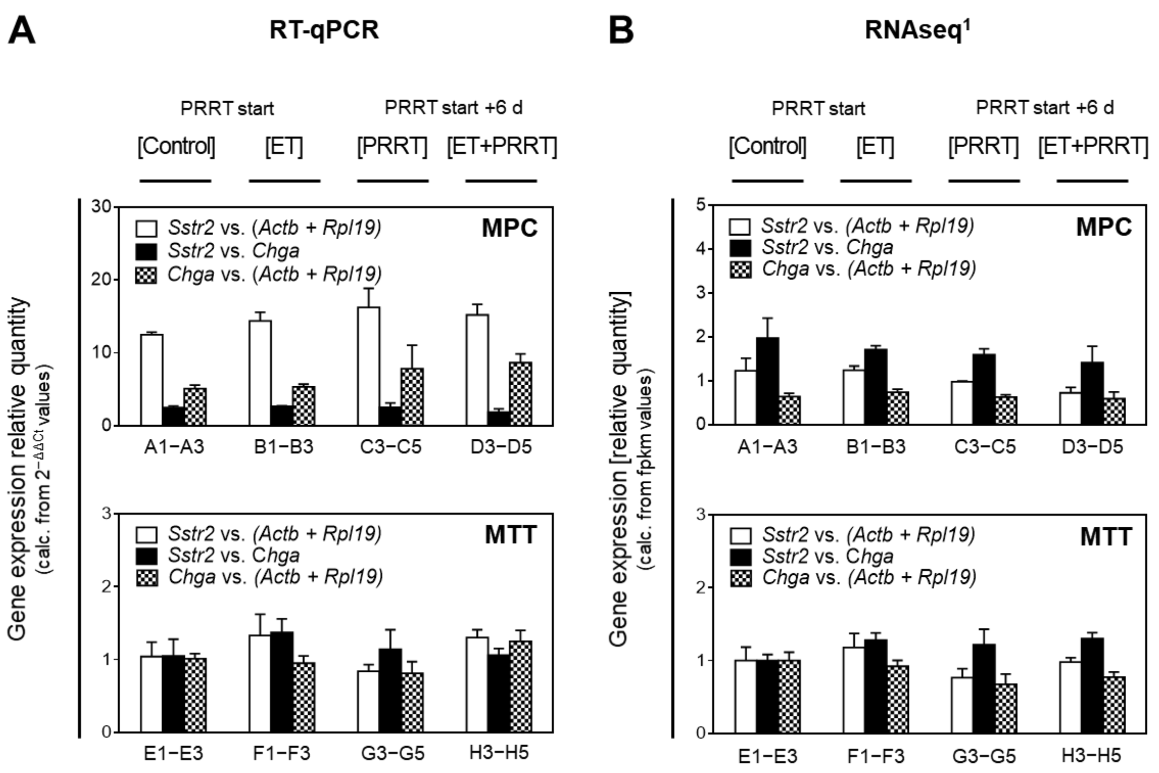

Figure S 8: Comparison of RT-PCR and RNAseq in gene expression analyses of selected genes in MPC and MTT tumors responding to treatments; ET: treatment with VPA (250 mg/kg) and DAC (1 mg/kg) as combination doses on days −4 and −1; PRRT: treatment with [^{177}Lu]Lu-DOTA-TATE (70 MBq/animal, equivalent to 1.2 nmol) as a single dose on day 0 **(A)** Relative gene expression ratios calculated from $2^{-\Delta\Delta Ct}$ values measured using RT-qPCR; **(B)** Relative gene expression ratios calculated from fpkm values (fragments per kilobase million) measured using RNAseq; all data were normalized to the average of MTT [Controls]; [1] mRNA samples MPC and MTT tumors were analyzed in separate RNAseq runs

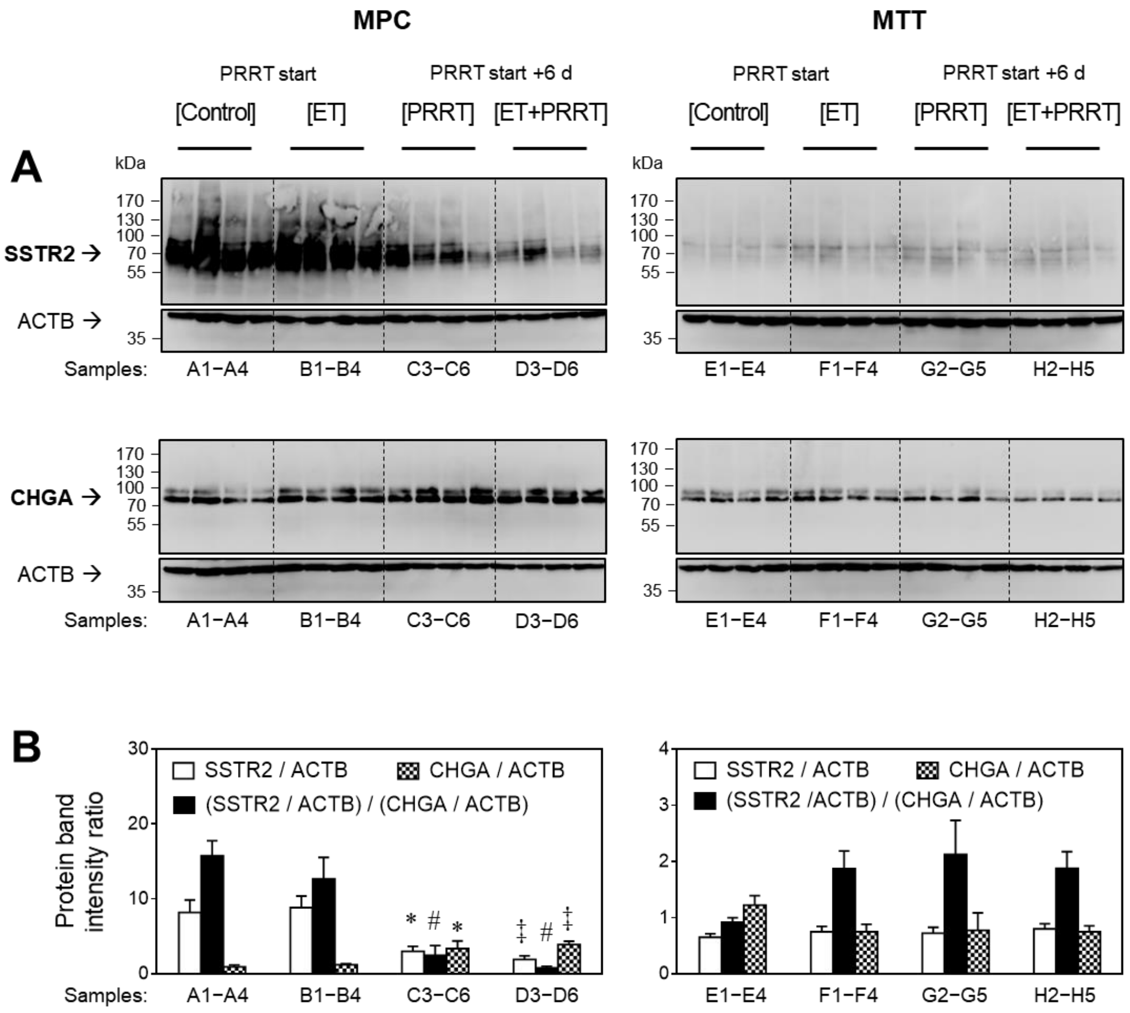

Figure S 9: Immunoblots analyzed for assessing SSTR2 and CHGA levels in tumors responding to treatments; ET: treatment with VPA (250 mg/kg) and DAC (1 mg/kg) as combination doses on days −4 and −1; PRRT: treatment with [^{177}Lu]Lu-DOTA-TATE (70 MBq/animal, equivalent to 1.2 nmol) as a single dose on day 0; **(A)** Total protein extracted from MPC tumors (14 µg/lane) and MTT tumors (24µg/lane) was separated using SDS polyacrylamide gel electrophoreses and blotted to PVDF membranes; immunodetection of SSTR2 and CHGA was done within the same experimental run on different membranes using identical concentrations of antibodies and developer substrate; **(B)** On each membrane, band intensities of the target proteins were normalized to ACTB as loading control; SSTR2/CHGA ratios were calculated from normalized target intensities; significance of differences: * $P < 0.05$; ‡ $P < 0.01$, # $P < 0.001$

2.5 Transcriptional responses of allograft tumors to epigenetic drugs and [^{177}Lu]Lu-DOTA-TATE – all genes and gene sets included

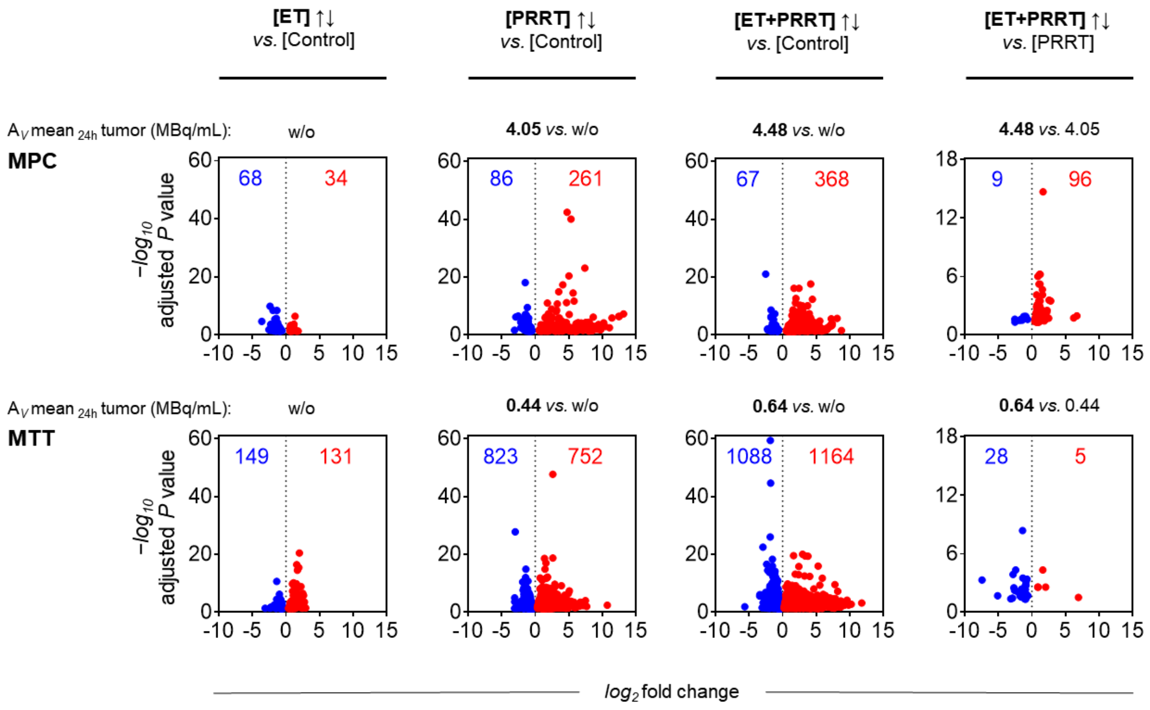

Figure S 10: Numbers of differentially expressed genes in MPC and MTT tumors in response to treatments; ET: treatment with VPA (250 mg/kg) and DAC (1 mg/kg) as combination doses on days −4 and −1; PRRT: treatment with [^{177}Lu]Lu-DOTA-TATE (70 MBq/animal, equivalent to 1.2 nmol) as a single dose on day 0; all protein-coding genes; $P_{adj} < 0.05$

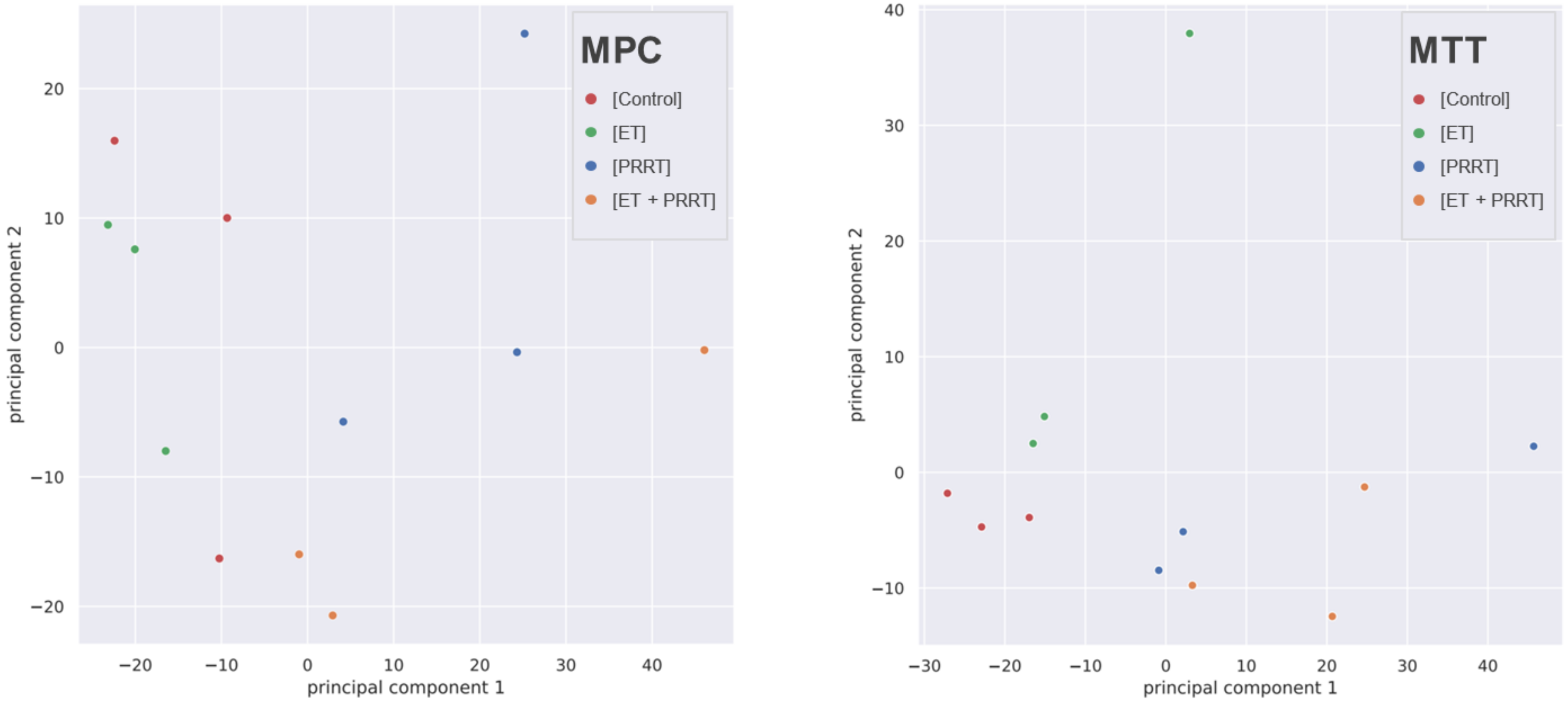

Figure S 11: Principle component analysis of gene expression in MPC and MTT tumors responding to treatments; all genes included

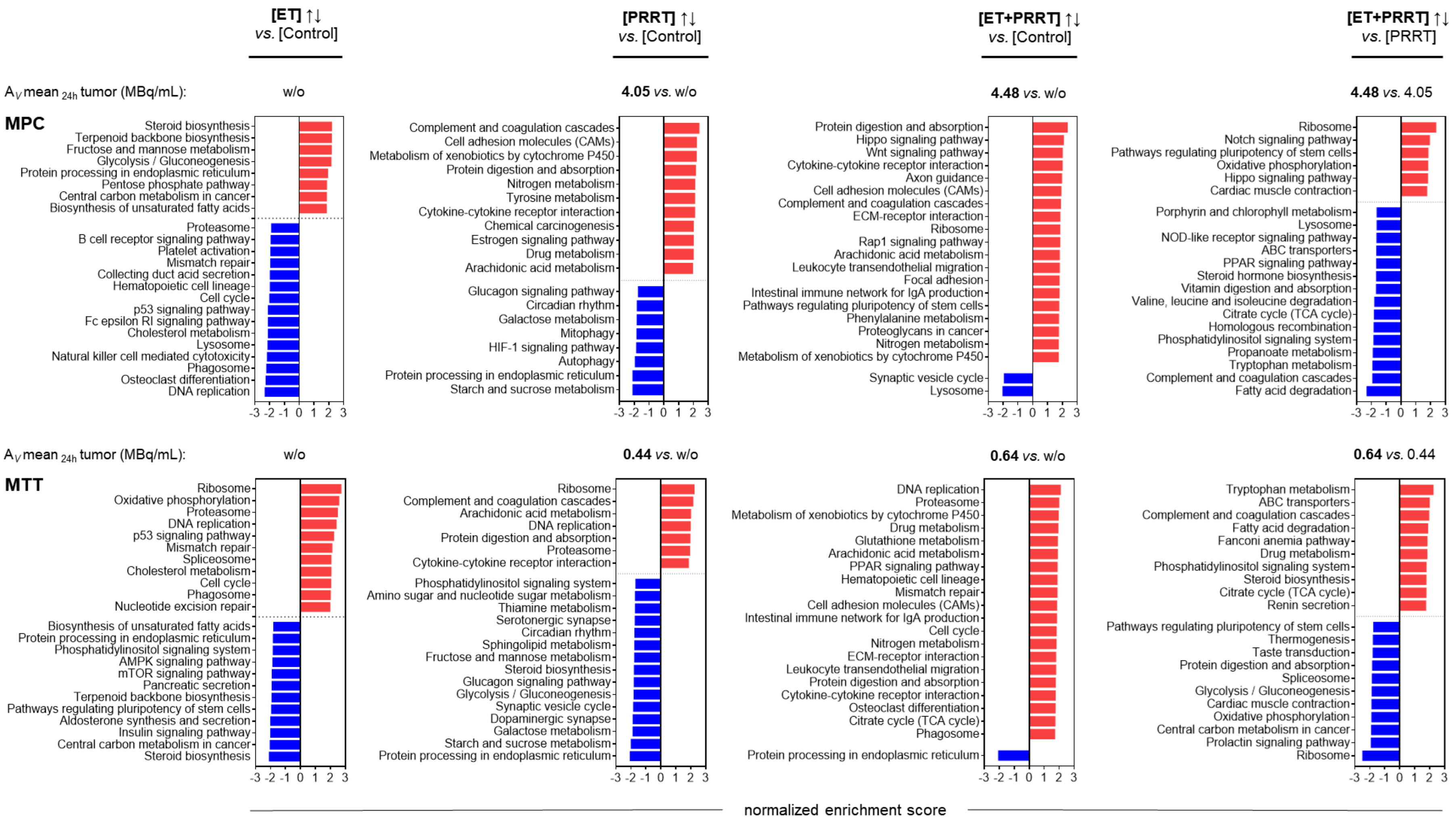

Figure S 12. Gene set enrichment analysis in MPC and MTT tumors − top-10 percent regulated gene sets in response to treatments; analysis based on KEGG pathway database; red bars: up-regulated pathways; blue bars: down-regulated pathways; pathways related to specific diseases have been excluded from the analysis; fdr < 0.25

2.6 *Transcriptional responses of allograft tumors to epigenetic drugs and [^{177}Lu]Lu-DOTA-TATE – pre-selected gene sets involved in cancer and radiation resistance*

Since the top-regulated gene sets in MPC and MTT tumors showed a number of pathways attributed to treatment-associated tissue damage and infiltration of leukocytes, a more specific pathway analysis was performed focusing on 39 pre-selected gene sets known to be involved in cancer and radiation resistance (see also Additional Methods 1.7).

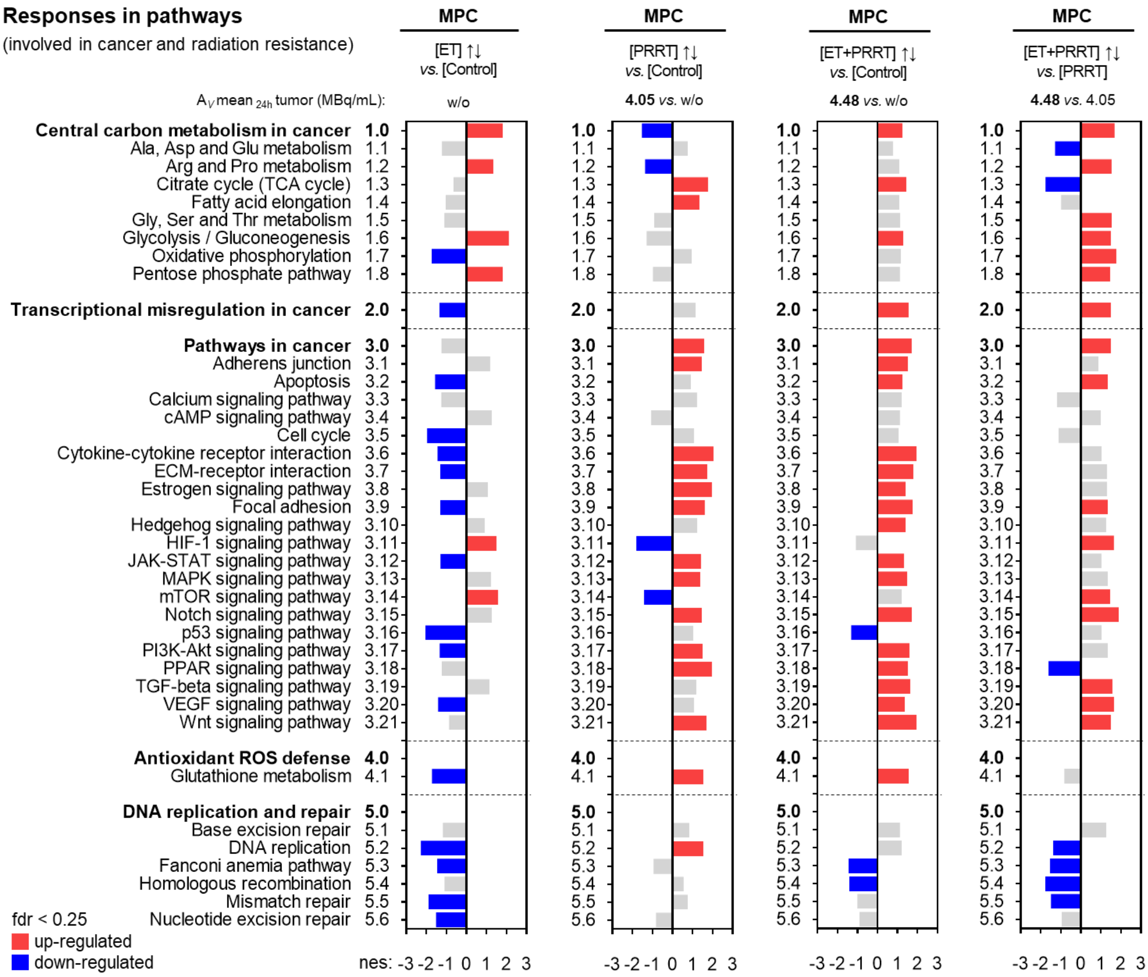

Figure S 13: Gen set enrichment in MPC tumors − treatment responses in pre-selected gene sets involved in cancer and radiation resistance; analysis based on KEGG pathway database; colored bars: fdr < 0.25; grey bars: fdr > 0.25

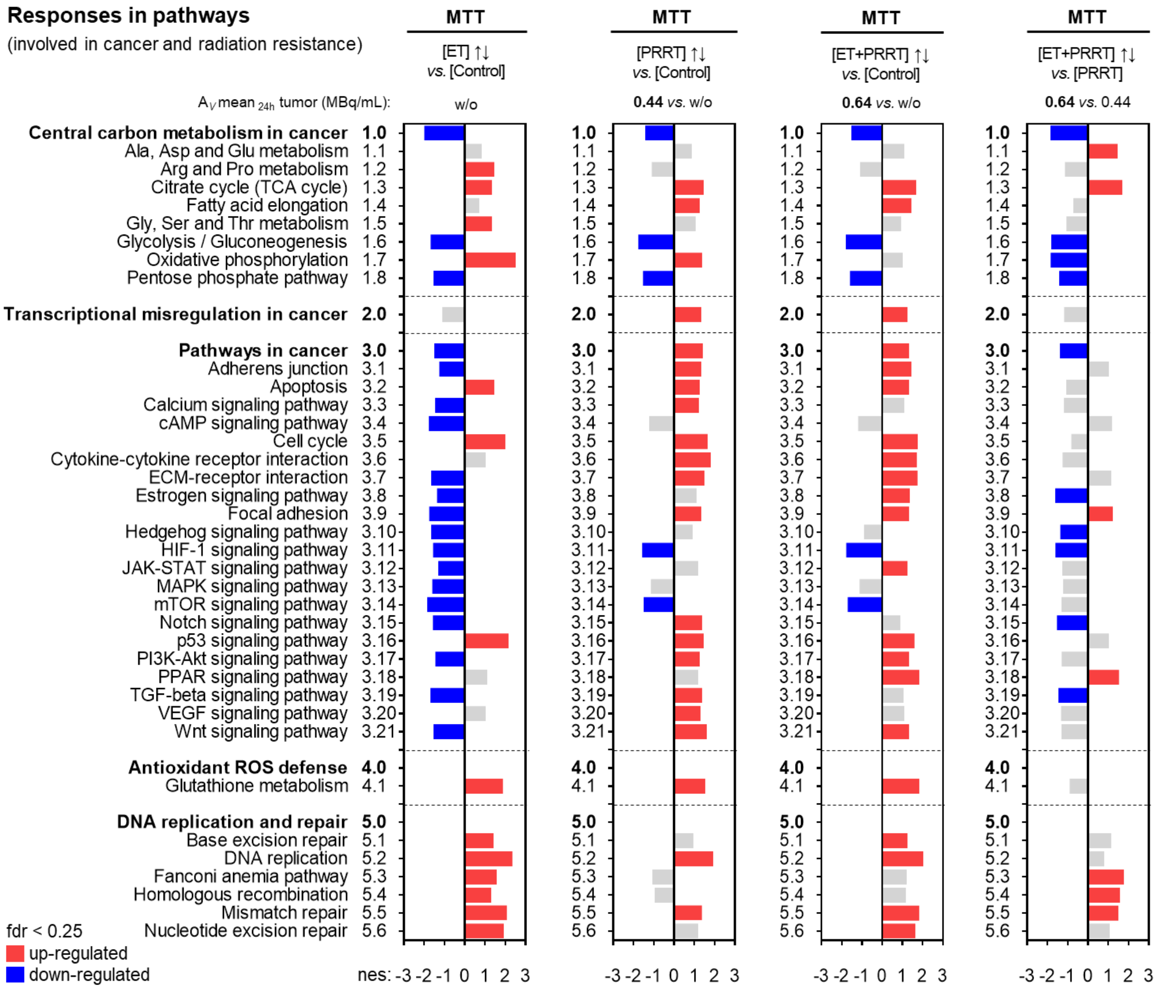

Figure S 14: Gen set enrichment in MTT tumors − treatment responses in pre-selected gene sets involved in cancer and radiation resistance; analysis based on KEGG pathway database; colored bars: fdr < 0.25; grey bars: fdr > 0.25

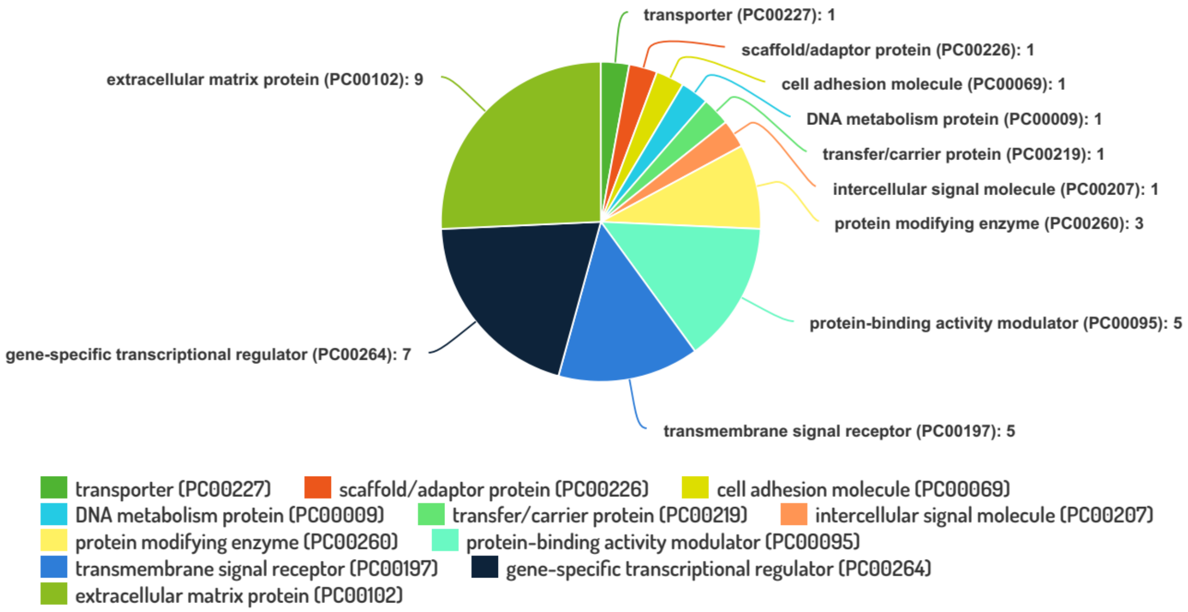

Figure S 15: Protein classes encoded by leading-edge genes in MPC tumors responding specifically to [^{177}Lu]Lu-DOTA-TATE; extracted from enrichment gene sets involved in cancer and radiation resistance; PANTHER gene list analyses based on gene ontology classification; $P_{adj} < 0.05$

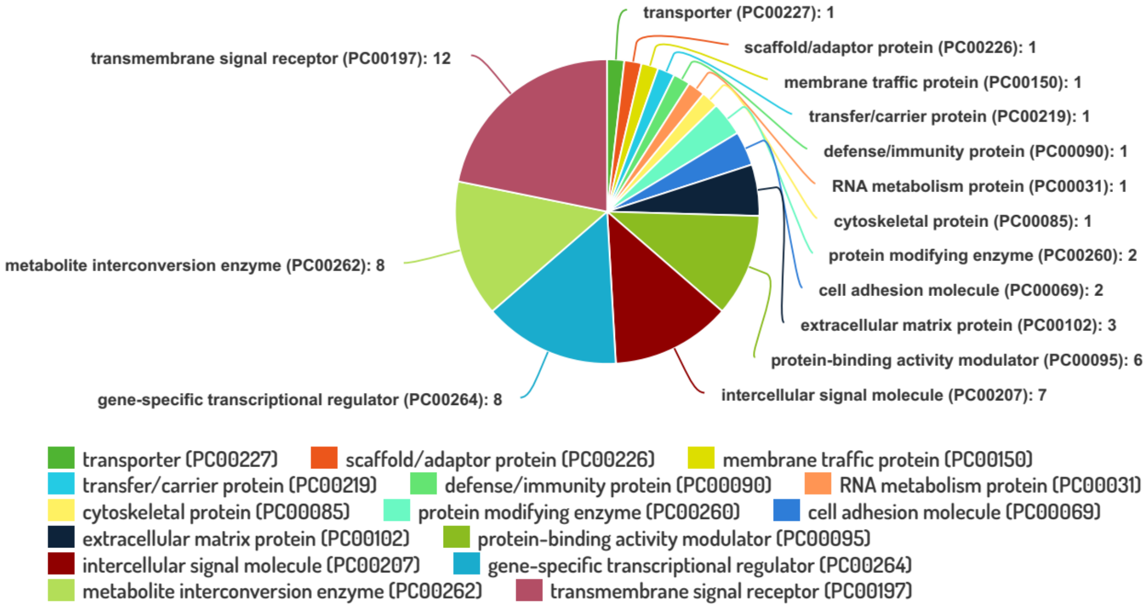

Figure S 16: Protein classes encoded by leading-edge genes in MTT tumors responding specifically to [^{177}Lu]Lu-DOTA-TATE; extracted from enrichment gene sets involved in cancer and radiation resistance; PANTHER gene list analyses based on gene ontology classification; $P_{adj} < 0.05$

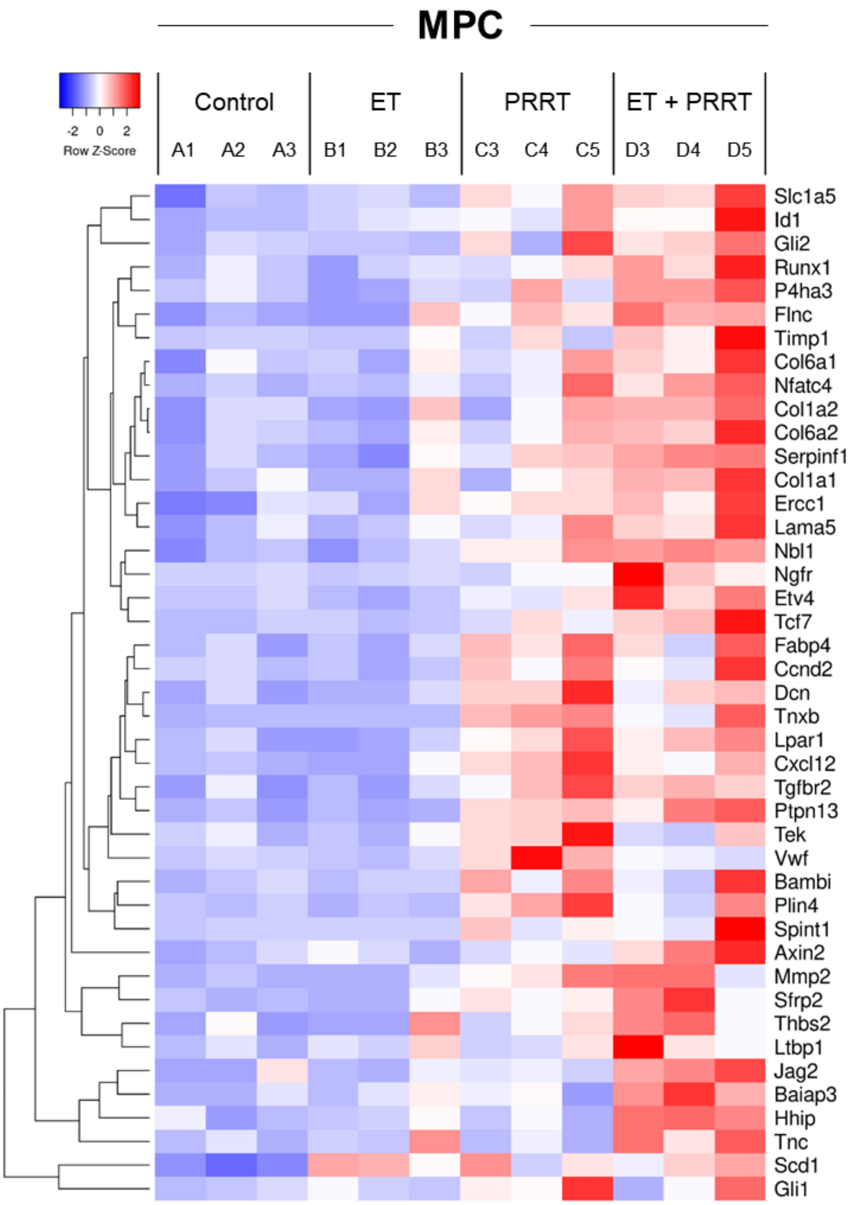

Figure S 17: Upregulated leading-edge genes in MPC tumors responding specifically to [^{177}Lu]Lu-DOTA-TATE; extracted from enrichment gene sets involved in cancer and radiation resistance; row clustering: average linkage of distances determined from Spearman rank correlation; $P_{adj} < 0.05$

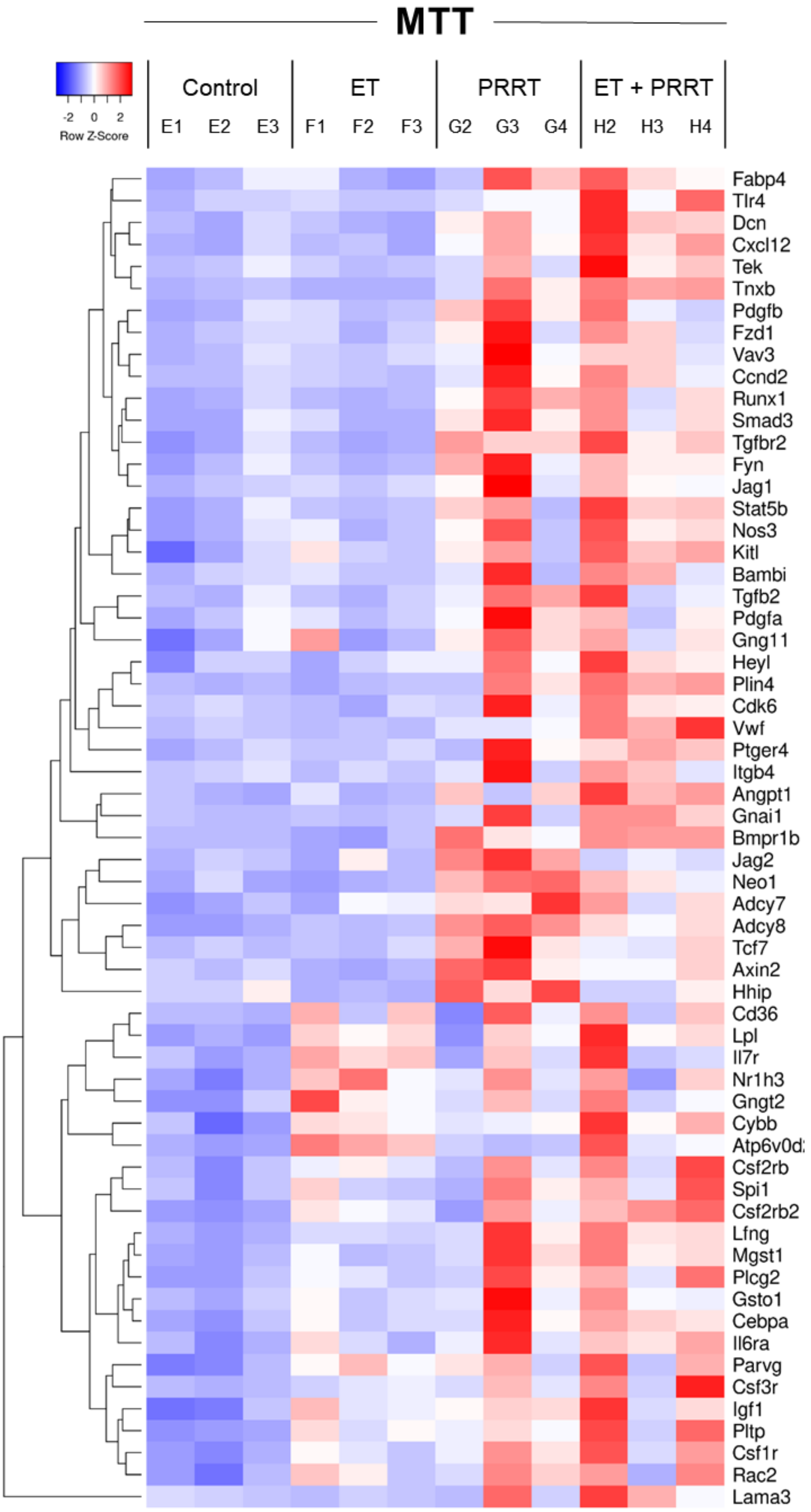

Figure S 18: Upregulated leading-edge genes in MTT tumors responding specifically to [^{177}Lu]Lu-DOTA-TATE; extracted from enrichment gene sets involved in cancer and radiation resistance; row clustering: average linkage of distances determined from Spearman rank correlation; $P_{adj} < 0.05$

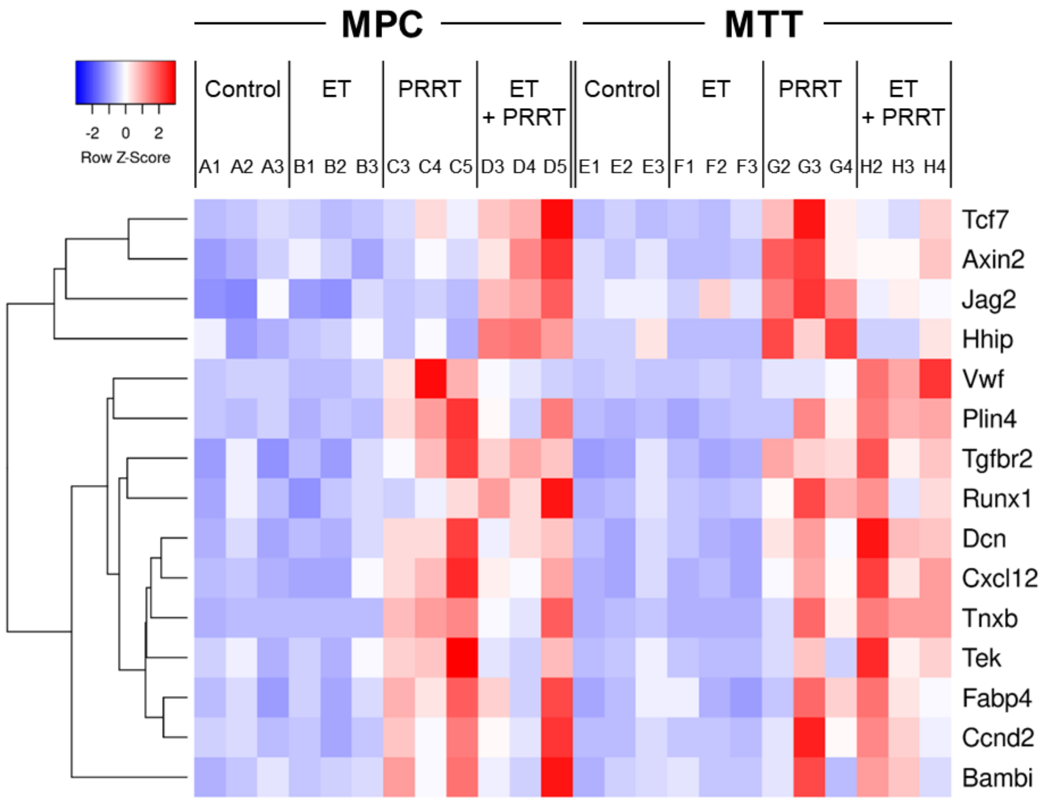

Figure S 19: Upregulated leading-edge genes shared between MPC and MTT tumors responding specifically to [^{177}Lu]Lu-DOTA-TATE; extracted from enrichment gene sets involved in cancer and radiation resistance; row clustering: average linkage of distances determined from Spearman rank correlation; $P_{adj} < 0.05$
